# SLC1A5 is a novel biomarker associated with ferroptosis and the tumor microenvironment: a pancancer analysis

**DOI:** 10.18632/aging.204911

**Published:** 2023-08-10

**Authors:** Peng Chen, YongAn Jiang, JiaWei Liang, JiaHong Cai, Yi Zhuo, HengYi Fan, RaoRao Yuan, ShiQi Cheng, Yan Zhang

**Affiliations:** 1 Department of Neurosurgery, The Second Affiliated Hospital of Nanchang University, Nanchang 330006, Jiangxi, P.R. China; 2 Department of Medical, Nanchang University, Nanchang 330006, Jiangxi, P.R. China

**Keywords:** solute carrier family 1 member 5 (SLC1A5), pancancer, prognostic biomarker, immunotherapy response, ferroptosis

## Abstract

Solute carrier family 1 member 5 (SLC1A5) is a member of the solute carrier (SLC) superfamily of transporters and plays an important role in tumors as a key transporter of glutamine into cells. However, the relationship between SLC1A5, which is involved in immune regulation, and immune cell infiltration in the tumor microenvironment has not been elucidated, and the relationship between SLC1A5 and ferroptosis is rarely reported. Therefore, we comprehensively analyzed the expression level of SLC1A5 across cancers and compared it with that in normal tissues. Then, the relationship between SLC1A5 expression and the tumor immune microenvironment was analyzed by single-cell analysis, gene set enrichment analysis (GSEA), and Tumor Immune Estimation Resource (TIMER). Next, the correlations of the SLC1A5 expression level with immunotherapy response, immunomodulator expression, tumor mutation burden (TMB) and microsatellite instability (MSI) were evaluated. Finally, *in vitro* experiments verified that SLC1A5 participates in ferroptosis of glioma cells to regulate tumor progression. Our results indicated that SLC1A5 is aberrantly expressed in most cancer types and closely associated with prognosis. The GSEA results showed that SLC1A5 is involved in immune activation processes and closely related to the infiltration levels of different immune cells in different cancer types. Upon further investigation, we found that SLC1A5 is a suppressor of ferroptosis in glioma, and SLC1A5 knockdown inhibited the proliferation and migration of glioma cells *in vitro*. In conclusion, we conducted a pancancer analysis of SLC1A5, demonstrated its role as a prognostic biomarker in cancer patients and explored its potential biological functions.

## INTRODUCTION

Cancer, as the second leading cause of global mortality, has emerged as an enduring public health challenge worldwide. There were 19.29 million cancer cases and 9.96 million cancer-related deaths worldwide in 2020 [1]. Among the deadliest cancers, brain cancer claims the lives of over 10 million individuals annually [2, 3]. Glioblastoma (GBM), as the most common primary infiltrating brain tumor, accounts for approximately 25% of central nervous system tumors [4]. According to the World Health Organization (WHO) tumor grading criteria, GBM represents the most malignant type among gliomas, classified as WHO grade IV [3, 5]. GBM is well known for its highly invasive nature and frequent recurrence, and it has a high mortality rate [6]. The commonly employed treatment modalities for GBM include radiation therapy, chemotherapy, and surgery [6, 7]. If left untreated, it can lead to death within 6 months [6]. Even with standard treatment, patients who survive 2 years after diagnosis are considered long-term survivors [6, 7]. However, over the past two decades, therapeutic advancements for GBM have been minimal [7]. Notably, cancer immunotherapy, which has demonstrated remarkable efficacy across a broad spectrum of cancers, is now emerging [8, 9]. By manipulating the immune system, immunotherapy can counteract tumor immune suppression, resulting in sustained antitumor activity with reduced side effects [6, 8–12]. Nevertheless, a significant proportion of patients develop resistance to immunotherapy [13]. Consequently, the exploration of novel immunotherapy markers or immunomodulatory genes could facilitate the development of more precise immunotherapy regimens for glioma patients.

Immunotherapy approaches investigated in GBM include vaccine therapy, immune checkpoint blockade, oncolytic virus therapy, and chimeric antigen receptor T-cell (CAR-T) therapy [8]. However, in some clinical trials, immunotherapy targeting GBM has not achieved the expected success. A phase III randomized clinical trial targeting the EGFRvIII antigen with the peptide vaccine rindopepimut found no significant improvement in overall survival in EGFRvIII-positive GBM patients [14]. Another phase III randomized clinical trial involving the DCVax-L vaccine in combination with adjuvant chemoradiotherapy showed promising results with significant survival benefits for patients [15]. Oncolytic viruses exert their anticancer effects by activating antitumor immune responses. A phase I clinical trial using the recombinant poliovirus vaccine PVSRIPO in recurrent GBM patients demonstrated a 2-year survival rate of 21% in GBM patients [13]. On the other hand, clinical trials targeting immune checkpoints in GBM have primarily focused on PD-1/PD-L1 and/or CTLA-4, but early results have not been promising. For example, a phase III comparative trial of nivolumab and bevacizumab in treating recurrent GBM did not show an improvement in overall survival for patients [16]. Although pembrolizumab has been FDA-approved for patients with dMMR tumors (Lynch syndrome) or patients with MSI-high status, only a subset of GBM patients may benefit from it [17]. However, the challenges of immunotherapy in GBM extend beyond the treatment itself. Changes in the tumor microenvironment and the phenomenon of resistance also significantly impact the effectiveness of immunotherapy.

In addition to cancer cells, the tumor microenvironment (TME) includes various stromal cells, innate immune cells, and adaptive immune cells, which play either promoting or inhibitory roles in tumor initiation [10, 18]. To sustain the malignant phenotype of tumor cells, abundant energy support is essential [19, 20]. Therefore, multiple metabolic pathways, including glycolysis, one-carbon metabolism, the tricarboxylic acid cycle, and fatty acid synthesis, generate energy for tumor cell proliferation and metastasis [21]. However, the energy produced through these metabolic pathways is crucial for the proliferation of nontumor cells as well, including both antitumor and protumor immune cells [22–24]. The uneven distribution of nutrients and oxygen supply among different cells in distinct spatial locations results in metabolic heterogeneity within the TME, as cells adaptively utilize different nutrients [19, 20]. Consequently, when spatial and metabolic heterogeneity occurs within the TME, the inhibition of cancer cell proliferation and metastasis by inhibitors may also suppress or alter the effector activity of antitumor immune cells [21, 24, 25]. For a long time, immune therapies targeting PD-1 and CTLA-4 have been believed to activate antitumor T cells by impacting T-cell metabolism, but some patients develop resistance early in treatment [26]. Some researchers propose that metabolic adaptations to the TME may hinder the effectiveness of immune checkpoint blockade through “metabolic immune suppression,” impairing the metabolic reprogramming necessary for effector function [11, 27, 28]. Improving immune therapy and reducing resistance are of paramount importance, but a thorough understanding of immune cell functionality and nutrient uptake and utilization in cellular metabolism is necessary to overcome metabolic immune suppression within the TME.

In mammals, most metabolites are transported through transmembrane proteins in the solute carrier (SLC) family, most of which are expressed in immune and tumor cells [29, 30]. For instance, solute carrier family 1 member 5 (SLC1A5) is an important member of the amino acid carrier system and is mainly responsible for the transmembrane transport of glutamine and some neutral amino acids in a Na+-dependent manner [31, 32]. In addition, SLC1A5 is among the most widely studied transporters and participates in the progression of tumors by playing a role in proliferation, apoptosis and the cell cycle [30]. Recent studies have revealed that the SLC1A5 gene is highly expressed in various cancers, including breast cancer, lung cancer, and colorectal cancer [33–35]. Its expression has been associated with tumor progression and prognosis, suggesting its potential as a therapeutic target [33, 34, 36]. Relevant research on the preclinical drug V-2, which targets SLC1A5, has provided strong evidence that inhibiting SLC1A5 can effectively suppress tumor cell proliferation [37]. These findings suggest that SLC1A5 holds potential as a reliable and promising anticancer target. Although bioinformatics analysis has revealed a correlation between SLC1A5 expression and the prognosis of glioma patients [36], the precise role of SLC1A5 and its relationship with the immune status of glioma patients and immunotherapy remain unclear. Therefore, pancancer studies on the cancer biology of SLC1A5 are urgently needed. We first evaluated differential expression of SLC1A5 in pancancer and normal tissues and further investigated the RNA expression level of SLC1A5 in clinical glioma samples. In addition, we performed genomic alteration analysis, prognosis analysis, gene set enrichment analysis (GSEA), immune cell infiltration analysis and drug sensitivity analysis based on SLC1A5 expression across cancers. Finally, we demonstrated that SLC1A5 is a reliable pancancer prognostic biomarker as well as a robust biomarker for predicting the immunotherapy response.

## RESULTS

### Basic information on SLC1A5

**Figure 1:**
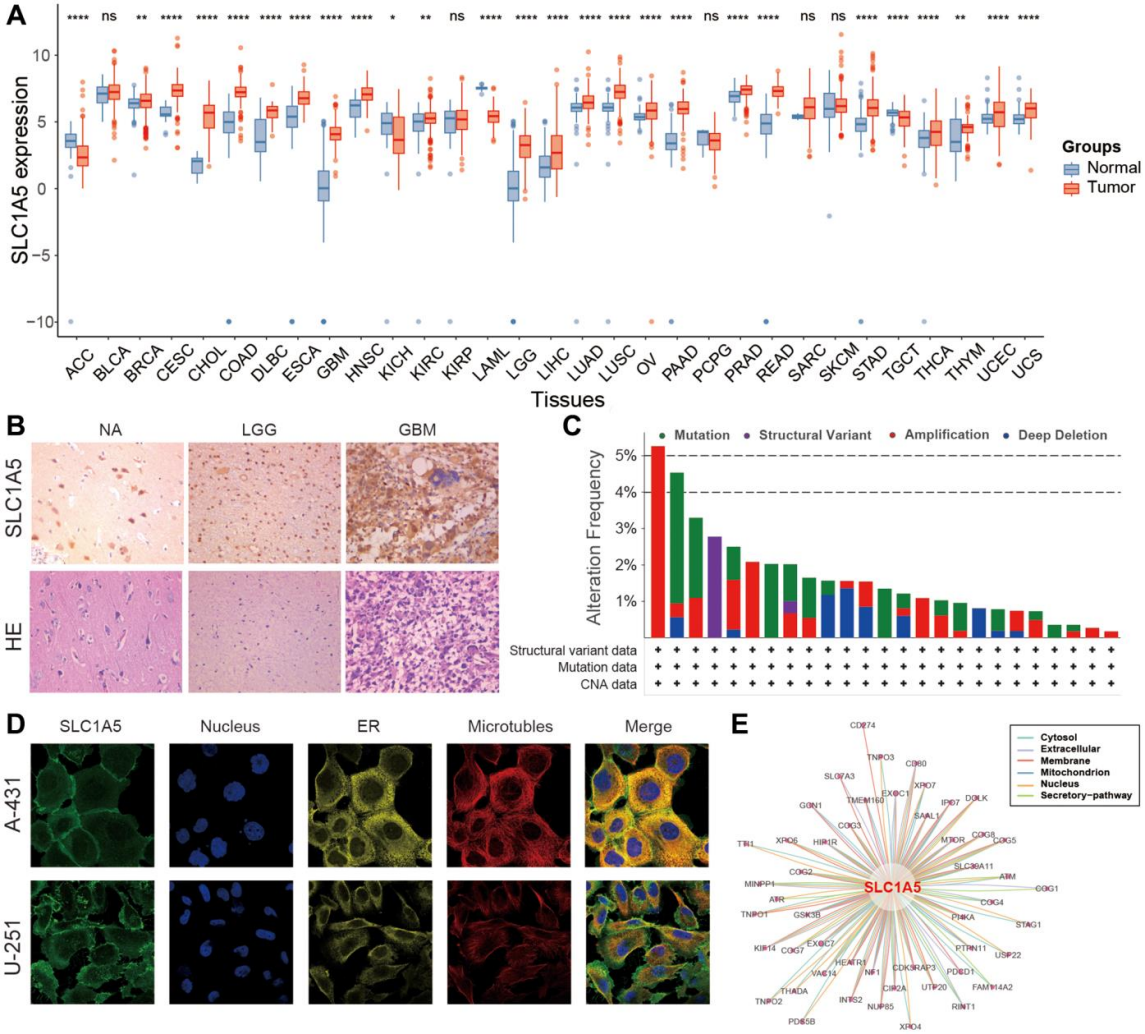
**Basic information on SLC1A5 across cancers.** (**A**) Expression levels of SLC1A5 in normal and cancerous tissues. (**B**) Immunohistochemistry and HE staining of SLC1A5 in normal brain and glioma tissues. (**C**) Alteration frequency of SLC1A5 across cancers based on the cBioPortal database. (**D**) Immunofluorescence images of SLC1A5 protein expression in the nucleus, endoplasmic reticulum (ER), and microtubules in A-431 and U-251 cells. (**E**) The protein‒protein interaction (PPI) network shows the proteins interacting with SLC1A5.

To first acquire basic information on SLC1A5 in cancer, we examined the distribution and expression level of SLC1A5 in tumors and normal tissues derived from different organs. The human organ map shows the pancancer distribution of SLC1A5 in the organs of male and female patients (Supplementary Figure 1A). Then, based on the expression data in TCGA and GTEx databases, the expression differences of SLC1A5 in different tumors and their corresponding normal tissues were analyzed. SLC1A5 was highly expressed in most tumors, including ACC, BRCA, CESC, CHOL, COAD, DLBC, ESCA, GBM, HNSC, KICH, KIRC, LAML, LGG, LIHC, LUAD, LUSC, OV, PAAD, PRAD, SKCM, STAD, TGCT, THCA, UCEC, and UCS (Figure 1A). Therefore, we performed immunohistochemical analysis, and as expected, SLC1A5 was significantly upregulated in GBM samples compared with LGG samples (Figure 1B and Supplementary Figure 1B). The analysis of genomic alterations of SLC1A5 showed that alterations of SLC1A5 are common across cancers; they are most UCS at a rate of 5%, and amplification is the most common alteration type (Figure 1C). In addition, immunofluorescence (IF) staining showed that SLC1A5 was mainly distributed and localized in the cell membrane of A-431 and U251 tumor cell lines (Figure 1D). Finally, a protein‒protein interaction network was constructed using protein interaction data obtained from the ComPPI online website, which described the subcellular localization of proteins closely related to SLC1A5 distributed in the cytoplasm, mitochondria, nucleus, extracellular, secretory pathway, and membrane (Figure 1E).

### Single-cell analysis of SLC1A5 in cancers

**Figure 2:**
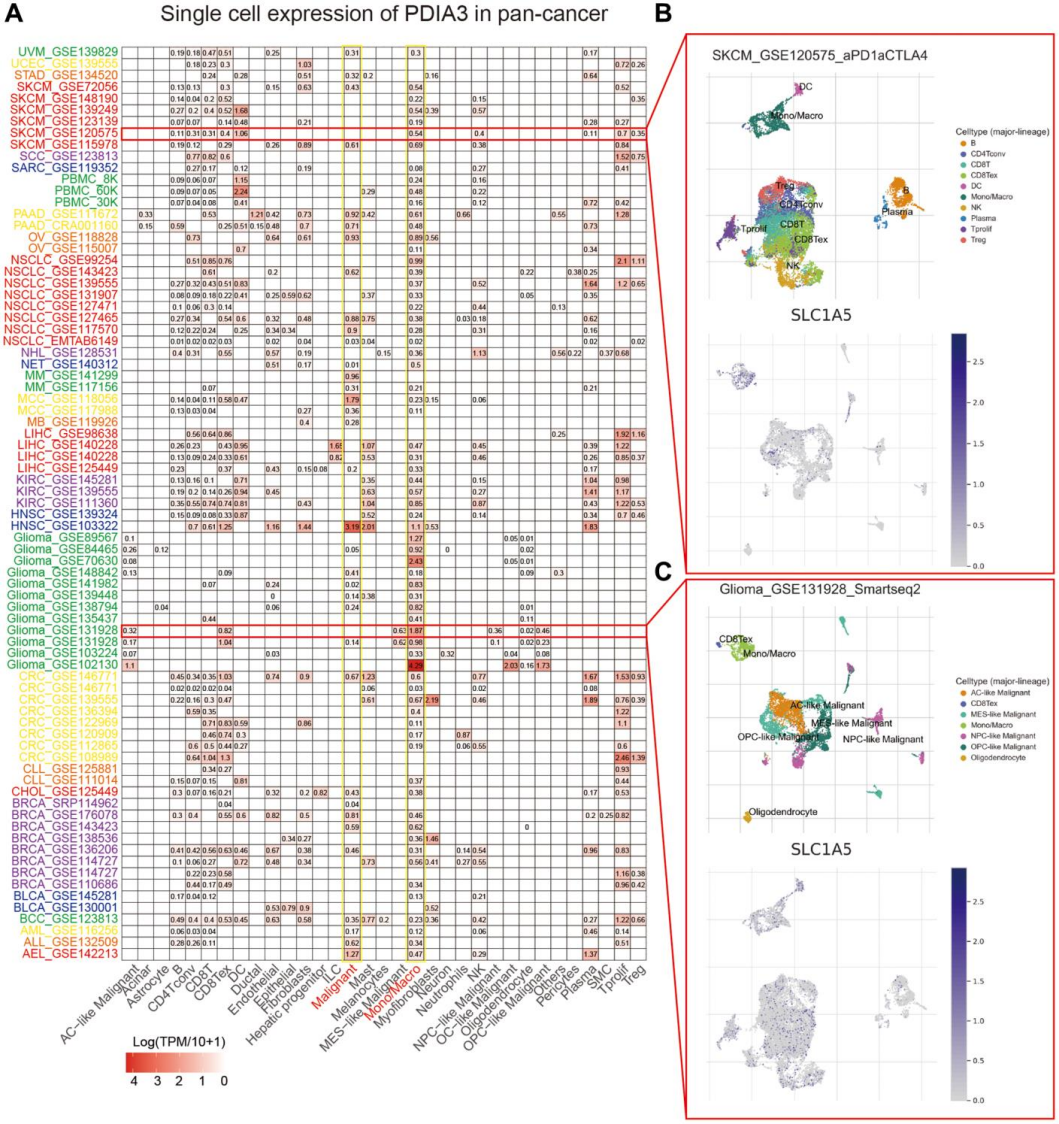
**Correlation of SLC1A5 with single-cell classes in the pancancer microenvironment.** (**A**) Heatmap showing the expression levels of SLC1A5 in 33 single cell types. (**B**) Scatterplot showing the GSE120575 dataset and the distribution of 10 different types of single cells. (**C**) Scatter plot showing the GSE131928 dataset and the distribution of 10 different types of single cells.

To gain insight into the major cell types expressing SLC1A5 in the tumor microenvironment, we performed single-cell analysis of SLC1A5 expression in 79 tumor single-cell databases. Based on the data obtained in the TISCH database, a heatmap was drawn to show the expression level of SLC1A5 in each cell type (including immune cells, stromal cells, malignant cells, and functional cells) in 79 single-cell datasets. The results indicated that across cancers, SLC1A5 was mainly expressed in immune cells (especially monocytes/macrophages) and malignant cells (Figure 2A). In the GSE120575 dataset containing 16,291 cells from 32 SKCM patients treated with immune checkpoint inhibitors, SLC1A5 was widely expressed in immune cell types in the SKCM microenvironment, such as T cells, dendritic cells, NK cells, monocytes or macrophages (Figure 2B). In the Glioma_GSE131928_Smartseq2 dataset, which contains 7,930 cells from 28 glioma patients, SLC1A5 was highly expressed in malignant cells and monocytes/macrophages in the glioma microenvironment (Figure 2C).

### Pancancer prognostic analysis of SLC1A5

**Figure 3:**
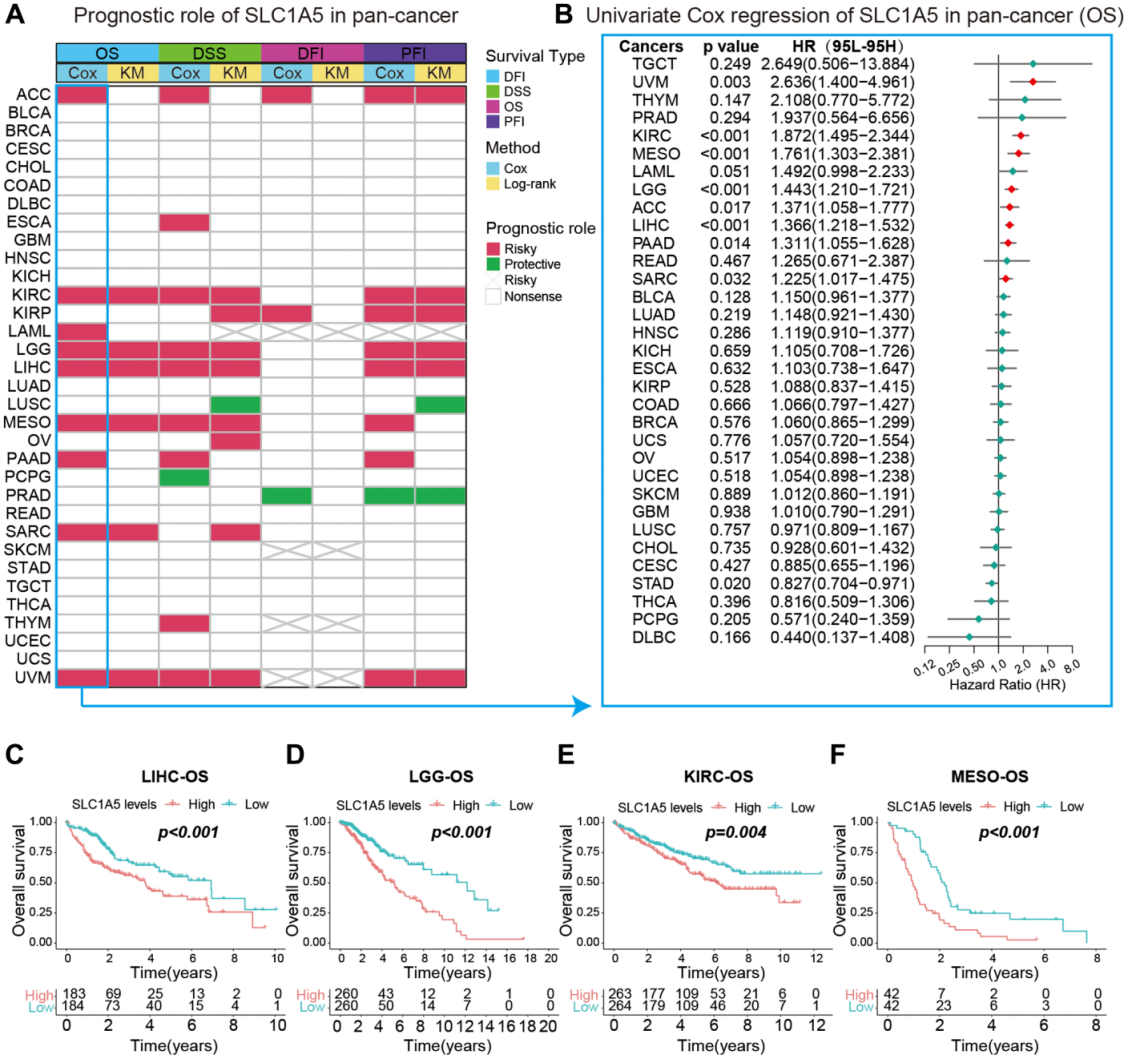
**Correlation analysis between SLC1A5 and the prognosis of different cancer patients.** (**A**) The summary of correlation analysis between SLC1A5 and patients’ overall survival (OS), disease-specific survival (DSS), disease-free interval (DFI) and progression-free interval (PFI) was analyzed by univariate Cox regression and Kaplan‒Meier. Red indicates that SLC1A5 is a risk factor for this type of cancer, and green indicates a protective factor. Only *p* values < 0.05 are shown. (**B**) Forest plot of the relationship between SLC1A5 and the prognosis of cancer patients analyzed by univariate Cox regression. Red markers indicate that SLC1A5 is a risk factor for this cancer type. (**C**–**F**) Kaplan‒Meier overall survival curves of SLC1A5 in LIHC (**C**), LGG (**D**), KIRC (**E**) and MESO (**F**).

For the survival landscape of SLC1A5 in pancancer, we showed the prognostic analysis of SLC1A5 with a heatmap, and the results showed that SLC1A5 is associated with the prognosis of most cancer types (BLCA, BRCA, CESC, CHOL, COAD, DLBC, GBM, HNSC, KICH, READ, SKCM, STAD, TGCT, THCA, UCEC, and UCS) (Figure 3A). The OS results showed that SLC1A5 was a protective factor for ACC, KIRC, LAML, LGG, LIHC, MESO, PAAD, SARC, and UVM. Since OS patient outcomes include noncancer mortality events, we used DSS to evaluate the association between SLC1A5 and patient cancer survival time. SLC1A5 was a protective factor in terms of DSS in LUSC and PCPG patients, but the trends for OS and DSS were consistent for all other cancer types. The DFI and PFI results were also examined to fully demonstrate that SLC1A5 is a risk factor for most cancer types and is significantly associated with the prognosis of cancer patients.

To further understand the prognostic potential of SLC1A5, after obtaining the OS data of 32 cancers in TCGA, the prognostic value of SLC1A5 in each cancer was analyzed using univariate Cox regression. The forest plot shows that high expression of SLC1A5 predicted shorter OS time in UVM (HR = 2.635 (95% CI, 1.400 to 4.960), *p* = 0.002), KIRC (HR = 1.871 (95% CI, 1.494 to 2.343), *p* < 0.001), MESO (HR = 1.761 (95% CI, 1.302 to 2.381), *p* < 0.001), LGG (HR = 1.443 (95% CI, 1.210 to 1.720), *p* < 0.001), LIHC (HR = 1.365 (95% CI, 1.217 to 1.531), *p* < 0.001), and PAAD (HR = 1.310 (95% CI, 1.055 to 1.628), *p* < 0.001) (Figure 3B). Kaplan‒Meier analysis was used to analyze the relationship between SLC1A5 expression and patient survival, and the results showed that high expression of SLC1A5 was associated with a poor prognosis in patients with KIRC (*p* < 0.004), LGG (*p* < 0.001), LIHC (*p* < 0.001), MESO (*p* < 0.001), STAD (*p* < 0.032), and UVM (*p* < 0.009) (Figure 3C–3F).

### GSEA of SLC1A5 in pancancer

**Figure 4:**
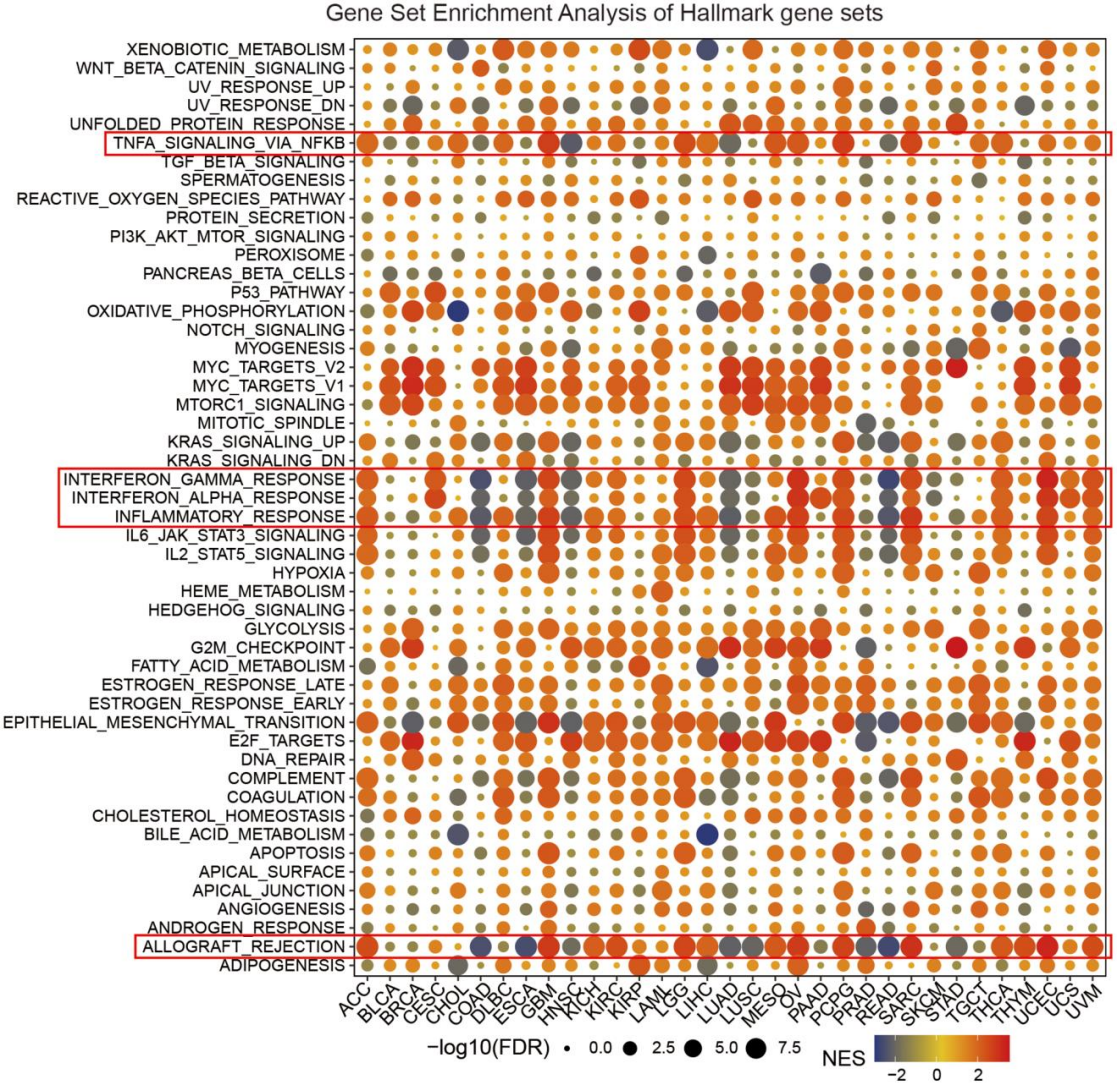
**Gene set enrichment analysis (GSEA) of the SLC1A5 gene set across cancers.** The size of the circle indicates the FDR value of the enriched element in each cancer, and the color indicates the normalized enrichment score (NES).

GSEA was performed based on the differentially expressed genes between the low-SLC1A5 subgroup and the high-SLC1A5 subgroup to evaluate the cancer characteristics related to SLC1A5 in each cancer type. To present the results of the GSEA in an intuitive and explicit way, a bubble diagram was used. As shown in Figure 4, SLC1A5 expression was strongly associated with immune-related pathways, such as TNFA signaling via NFKB, IFN-α response, IFN-γ response, and inflammatory response, especially in ACC, GBM, LGG, OV, PCPG, SARC, THCA, and UCEC. In addition, the E2F target pathway was enriched in BRCA, HNSC, LUAD, MESO, OV, PAAD, STAD, and THYM. E2F transcription factors have been reported to play an important role in preventing tumorigenesis by strictly controlling the cell cycle, maintaining genome integrity, and responding to replication stress and DNA damage. In addition, epithelial-mesenchymal transition, oxidative phosphorylation, unfolded protein response, MYC targets, and MTORC1 signaling are closely related to the expression of SLC1A5 in cancer. In conclusion, the above results indicate that the expression of SLC1A5 is closely related to the activation of the tumor immune microenvironment and the malignant phenotype of various cancers, which provides new clues for us to further explore the role of SLC1A5 in tumor progression.

### TIMER immune cell infiltration analysis

**Figure 5:**
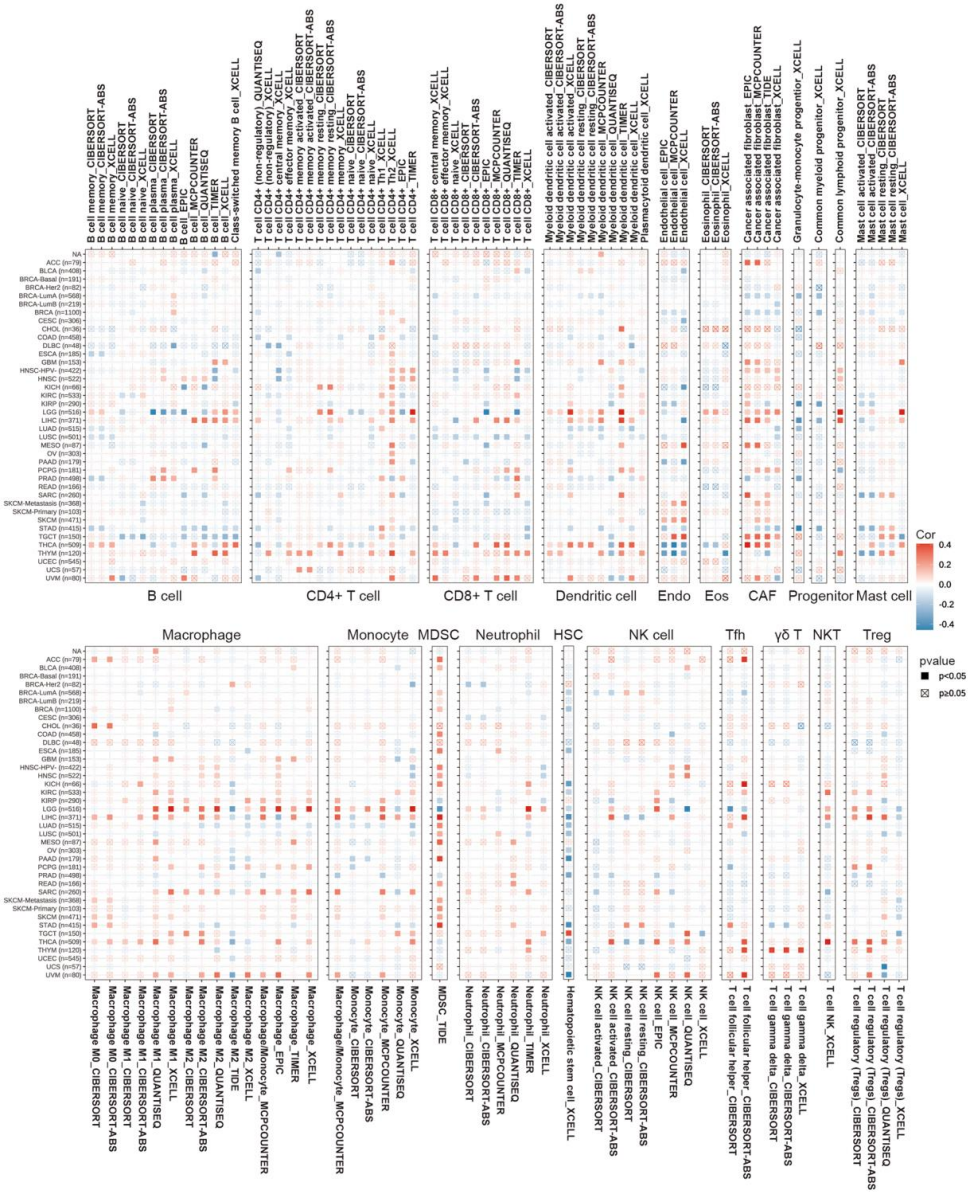
**Correlation of SLC1A5 expression with immune infiltration levels of B cells, CD4+ T cells, CD8+ T cells, dendritic cells, Endo, Eos, CAF, progenitor, mast cells, HSC, macrophages, monocytes, Tfh, γδT, NKT, regulatory T cells (Tregs), neutrophils, NK cells, and MDSCs in cancers.** Red and blue indicate positive and negative correlations, respectively.

Based on the indication from the GSEA results that SLC1A5 is closely related to cancer immunity, we investigated the correlation between the expression of SLC1A5 and immune cell infiltration across cancers. Spearman correlation analysis was performed using pancancer immune infiltration data from the TIMER2 database. The results showed the infiltration levels of CD4+ T cells, CAFs, lymphoid progenitors, myeloid progenitors, monocyte progenitors, Endos, Eos, HSCs, Tfhs, γ/δ T cells, NK T cells, Tregs, B cells, neutrophils, monocytes, macrophages, dendritic cells, NK cells, mast cells, and CD8+ T cells in different cancer types (Figure 5). In most cancers, the expression of SLC1A5 is positively correlated with the infiltration of macrophages, CD4+ T cells, CD8+ T cells, dendritic cells, monocytes, MDSCs, and CAFs and negatively correlated with the infiltration of monocyte, HSC and Endo progenitors. In addition, SLC1A5 was closely associated with monocyte, dendritic cell, CAF and macrophage infiltration in LGG. Given the significant correlation between SLC1A5 expression and macrophage infiltration, we further investigated the relationship between SLC1A5 expression and macrophage subtype infiltration. The results revealed a positive correlation between SLC1A5 expression and M2 macrophage infiltration in LGG, but there was no significant correlation with M1 macrophages. Related reports have noted that the accumulation of tumor-associated M2 macrophages composed of brain-resident microglia and monocyte-derived macrophages is associated with a poor prognosis in patients with glioma [38]. Therefore, the important role of immune cells in tumor therapy cannot be ignored. Taken together, our results suggest that SLC1A5 affects the progression, treatment and prognosis of cancer patients by regulating immune cells.

### Relationships between SLC1A5 and immune regulators, TMB, and MSI

**Figure 6:**
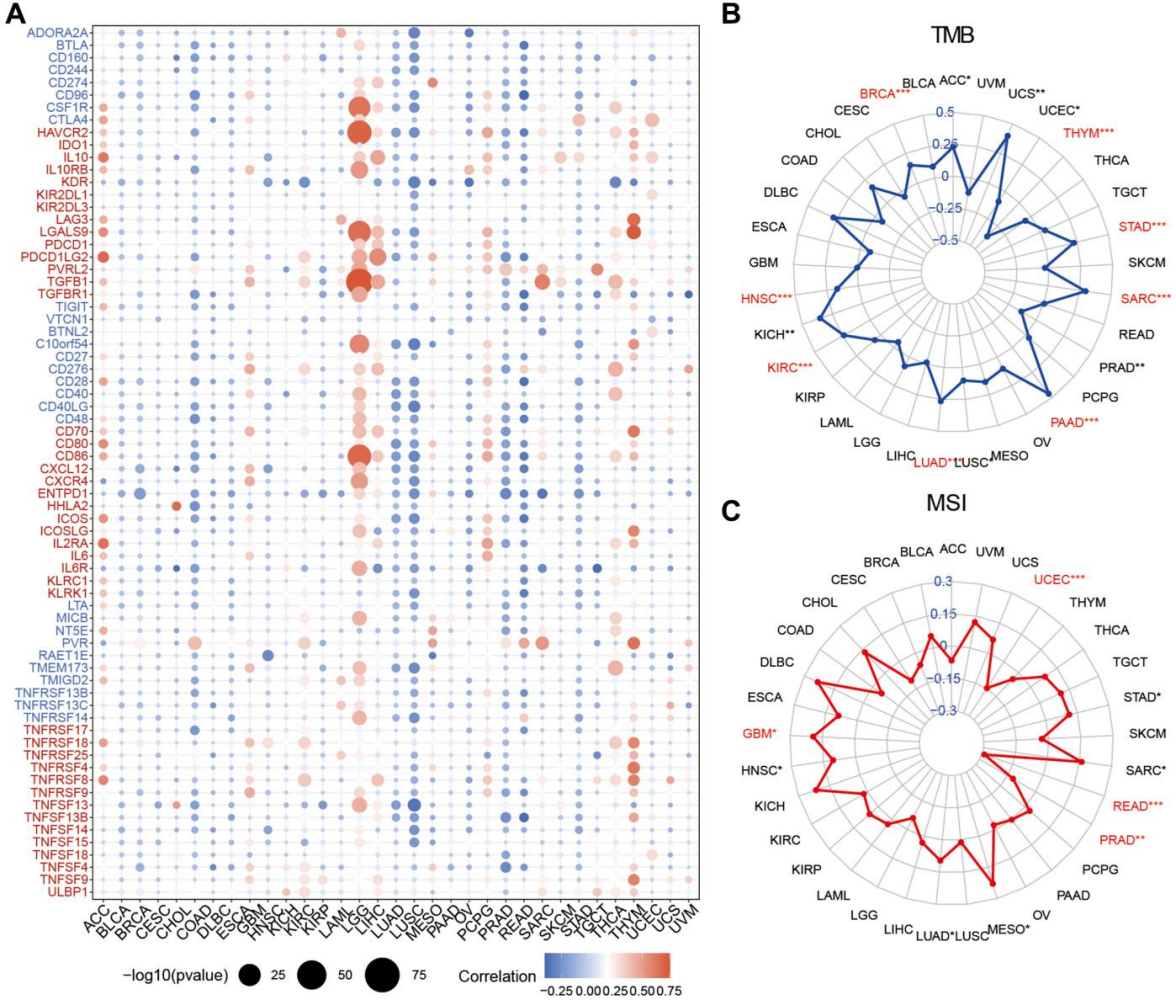
**Relationship between SLC1A5 and immunotherapy-independent efficacy predictive biomarkers.** (**A**) The Spearman correlation heatmap shows that the expression level of SLC1A5 is correlated with 46 immunostimulators and 24 immunoinhibitors. Red indicates a positive correlation and blue indicates a negative correlation. (**B**) The relationship between SLC1A5 expression level and tumor mutation burden (TMB) in cancer patients. (**C**) The relationship between the SLC1A5 expression level and microsatellite instability (MSI) in cancer patients. The labeled asterisk indicates the statistical *p* value (^*^p < 0.05, ^**^p < 0.01, ^***^p < 0.001).

The correlations of the expression levels of 70 genes encoding immunomodulators, including 46 genes encoding immunostimulators and 24 genes encoding immunoinhibitors, and those of SLC1A5 across cancers is shown in Figure 6A. We found that SLC1A5 was positively correlated with most immunomodulators in ACC, LGG, and THYM but negatively correlated with LUAD, LUSC, PRAD, and READ. Recently, immune checkpoint inhibitors (ICIs) have been highlighted as potential clinical treatments, so we next analyzed the association between SLC1A5 expression and TMB and MIS. Our results showed that SLC1A5 expression had a strong positive association with TMB in BRCA, HNSC, KIRC, LUAD, PAAD, SARC, STAD, and THYM (Figure 6B). In addition, the correlation between SLC1A5 expression and MSI was positive in GBM, HNSC, LUAD, MESO, and SARC and negative in READ and UCEC (Figure 6C). Our results demonstrate that SLC1A5 can predict the efficacy of ICIs in the treatment of various cancers.

### The predictive role of SLC1A5 in cancer immunotherapy

By targeting and inhibiting the actions of immune checkpoint molecules, immune checkpoint blockade (ICB) therapy can activate the immune system to mount an attack against the tumor. PD-1/PD-L1 and CTLA-4 are the most commonly targeted molecules. Clinical trials have demonstrated the remarkable efficacy of immune checkpoint inhibitors across various tumor types, particularly melanoma, non-small cell lung cancer, renal cell carcinoma (RCC), and bladder cancer, among others [39]. Building upon the aforementioned findings, we further investigated the predictive role of SLC1A5 in the ICB therapy cohort. In the GBM-PRJNA482620 cohort, there was no statistically significant difference in the expression level of SLC1A5 between responders and nonresponders (Supplementary Figure 2A). However, high expression of SLC1A5 in patients treated with anti-PD1 was associated with a poorer prognosis (Supplementary Figure 3A). In melanoma patients, it was observed that low expression levels of SLC1A5 in the GSE78220_anti-PD1 and GSE106128_DCs cohorts were significantly associated with a favorable response to ICB therapy (Supplementary Figure 2B and 2C). Furthermore, patients with low SLC1A5 expression exhibited improved prognosis (Supplementary Figure 3B and 3C). In contrast, in melanoma patients from the Nathanson_2017 cohort, it was observed that patients with low expression of SLC1A5 who received ai_CTLA-4 treatment had a worse prognosis (Supplementary Figures 2D and 3D). In RCC patients from the RCC_2020 cohort, contrasting ICB treatment responses and prognoses were observed in the high SLC1A5 expression group after ai-PD-1 and EVEROLIM treatment (Supplementary Figures 2E, 2F and 3E, 3F). These data confirmed that SLC1A5 expression levels in different cohorts of cancer patients were associated with varied immune checkpoint inhibitor treatment responses and prognoses, highlighting its potential as a predictive biomarker.

### Connectivity map (CMap) analysis of SLC1A5 in pancancer

**Figure 7:**
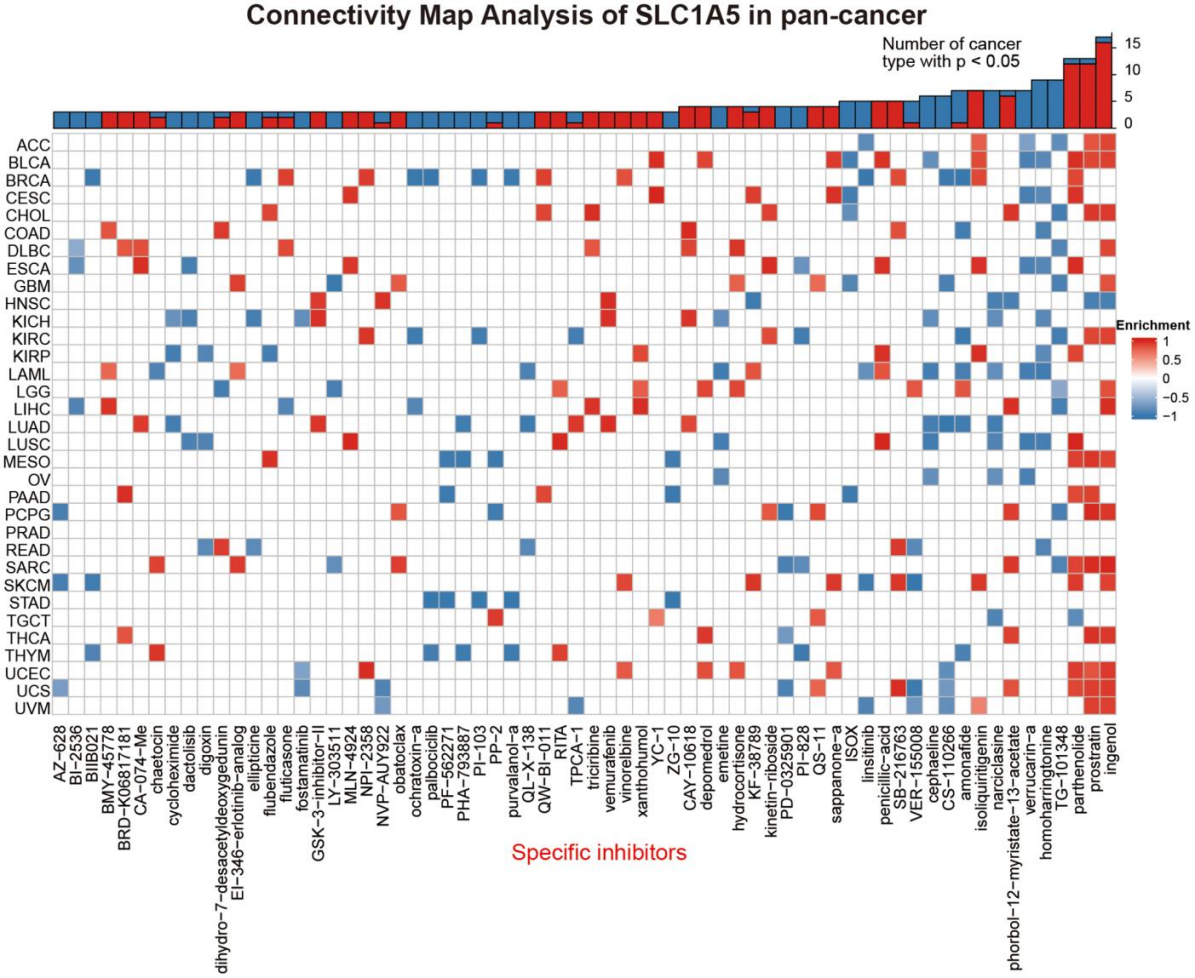
**The heatmap represents the enrichment score (positive in blue, negative in red) for each drug in each cancer in the CMap database.** Components or drugs are sorted from right to left with a decreasing number of enriched cancers.

The potential drugs and components targeting SLC1A5 in pancancer are shown as a heatmap in Figure 7. The heatmap shows the SLC1A5-related drug components that appear in 3 or more cancer types, and the enrichment parameters of each drug in pancancer are shown in Supplementary Table 1. As shown, ingenol-related genes were significantly enriched in 16 cancers, while prostratin- and parthenolide-related genes were enriched in 12 cancers. These drugs are generally related to the prevention and treatment of cancer; for example, ingenol plays an anticancer role by inhibiting the migration and invasion of colon cancer cells [40], and prostratin inhibits the proliferation of breast cancer cells [41]. The association of these drugs with SLC1A5 increased our interest in them. Our results showed that these drugs have only a small part in the treatment of cancer, and their roles and potential mechanisms in the occurrence and development of various cancers need to be further explored.

### SLC1A5 regulates the proliferation and migration of glioma cells

**Figure 8:**
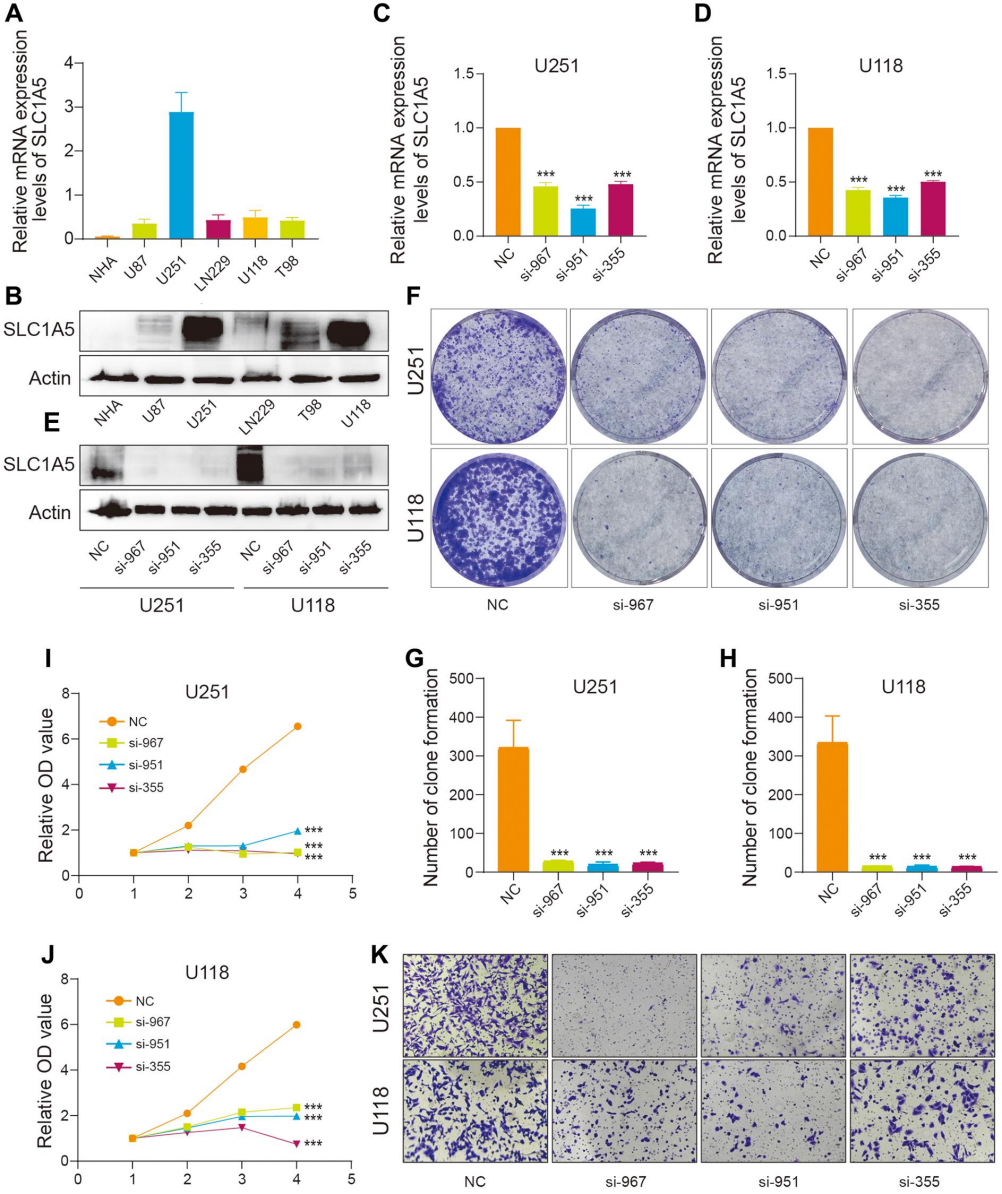
***In vitro* evidence that SLC1A5 is involved in the proliferation and migration of glioma cells.** (**A**) RNA and (**B**) protein expression levels of SLC1A5 in astrocytes and glioma cell lines. (**C**–**E**) RT‒qPCR and Western blotting verified the knockdown efficiency of SLC1A5 in U251 and U118 cell lines, respectively. (**F**) Colony formation experiments were performed to analyze the effect of SLC1A5 downregulation on the colony formation ability of U251 and U118 cell lines, and (**G**, **H**) statistical analysis was performed. (**I**, **J**) Cell proliferation ability was evaluated using the CCK-8 assay, and proliferation curves were plotted. (**K**) Cell migration ability was determined by transwell migration assay. The labeled asterisk indicates the statistical *p* value (^*^p < 0.05, ^**^p < 0.01, ^***^p < 0.001).

To determine the expression levels of SLC1A5 in tumor and normal cell lines, Western blot and RT‒qPCR analyses were performed. The results showed that the expression levels of SLC1A5 were higher in tumor cell lines than in normal cells (Figure 8A, 8B). Based on these results, the functions of SLC1A5 in the U251 and U118 cell lines were further investigated. The efficiency of SLC1A5 knockdown by siRNA was verified at the RNA and protein levels (Figure 8C–8E). Therefore, a loss-of-function experiment was conducted in which SLC1A5 was knocked down. The colony formation assay indicated that the suppression of SLC1A5 expression inhibited the proliferation of U251 and U118 cells (Figure 8F–8H). Moreover, the CCK-8 assay showed that cell growth was inhibited in the SLC1A5 knockdown group compared to the control group (Figure 8I, 8J). Furthermore, the transwell migration assay showed that SLC1A5 knockdown inhibited the migration of U251 and U118 cells (Figure 8K and Supplementary Figure 1C, 1D). Therefore, these results indicated that SLC1A5 promotes the proliferation and migration of glioma cells.

### SLC1A5 participates in glioma progression by inhibiting ferroptosis

**Figure 9:**
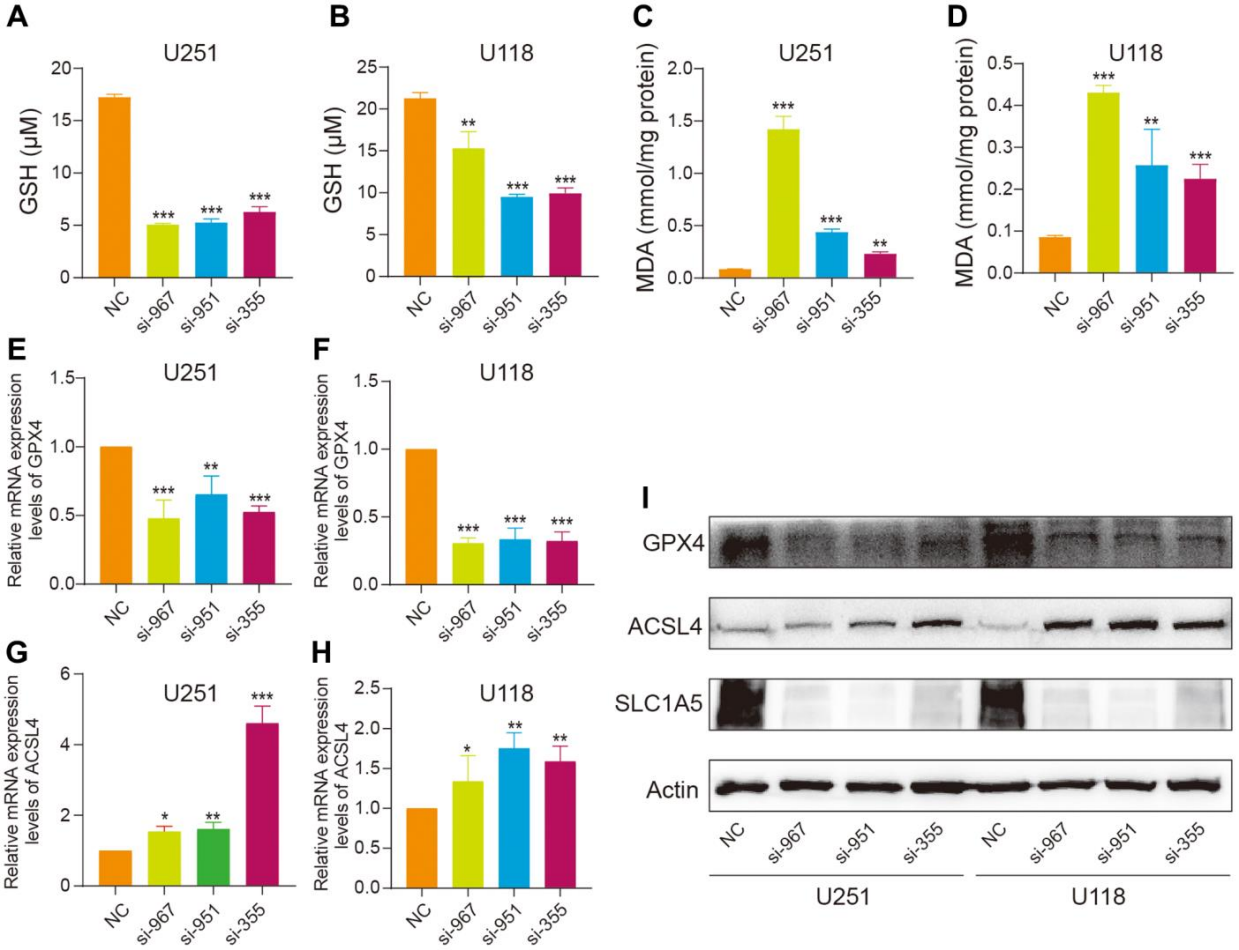
**SLC1A5 inhibits ferroptosis in glioma cells.** (**A**, **B**) The level of GSH was measured in U251 and U118 cells after SLC1A5 knockdown. (**C**, **D**) The level of MDA was measured in U251 and U118 cells after SLC1A5 knockdown. (**E**, **F**) The RNA expression levels of the ferroptosis gene GPX4 were detected in U251 and U118 cell lines after SLC1A5 knockdown. (**G**, **H**) The RNA expression levels of the ferroptosis gene ACSL4 in U251 and U118 cell lines were detected after SLC1A5 knockdown. (**I**) Western blotting was used to detect the protein expression levels of GPX4 and ACSL4 after SLC1A5 knockdown. The labeled asterisk indicates the statistical *p* value (^*^p < 0.05, ^**^p < 0.01, ^***^p < 0.001).

To study the effect of SLC1A5 knockdown on ferroptosis in glioma cells, we evaluated the level of ferroptosis in cells by measuring ferroptosis indexes with kits. GSH is an important antioxidant in cells that can scavenge lipid peroxides via glutathione peroxides and inhibit the occurrence of ferroptosis. As expected, knockdown of SLC1A5 resulted in a downregulation of GSH levels in U251 and U118 cells (Figure 9A, 9B). Since MDA is an end product of ferroptosis, we detected MDA and found that it was upregulated after SLC1A5 knockdown (Figure 9C, 9D). The RT‒qPCR and WB results showed that the expression of GPX4 was decreased and the expression of ACSL4 was increased in the siRNA group compared with the NC group (Figure 9E–9I). Thus, these data suggest that SLC1A5 promotes glioma progression by inhibiting ferroptosis in glioma cells.

## DISCUSSION

In the past few decades, ICB therapy has made great progress in the treatment of tumors, bringing hope for a cure in patients [42]. However, only a small fraction of cancer patients responds to immunotherapy due to the heterogeneity of the tumor microenvironment within each patient [43]. Finding biomarkers to predict a patient’s clinical response to immunotherapy will enable individualized treatment of patients. In this study, we found that SLC1A5 is a reliable pancancer prognostic biomarker and can effectively predict immunotherapy response. Therefore, our findings may provide clues for further studies to reveal the potential role of SLC1A5 in immunotherapy.

First, we combined the TCGA and GTEx databases to compare the mRNA expression levels of SLC1A5 between pancancer and normal tissues. The results showed that SLC1A5 was upregulated in most cancer types. Previous studies also indicated that SLC1A5 is upregulated in tumor tissues vs. nontumor tissues in patients with various tumor types, including lung cancer, liver cancer, and colon cancer [44]. Furthermore, it has been found that SLC1A5 is particularly upregulated in LGG and GBM tissue compared to normal brain tissue. Next, we collected glioma samples and performed RT‒qPCR to detect the expression of SLC1A5 and found that the expression of SLC1A5 in glioma tissues was higher than that in adjacent tissues. These findings suggest that SLC1A5 is commonly upregulated in various types of cancer. However, limited information is available regarding the association between SLC1A5 and patient prognosis in cancer. Therefore, we conducted a comprehensive investigation to further explore the relationship between SLC1A5 expression and patient outcomes in cancer.

Then, we evaluated the association of SLC1A5 with the prognosis of cancer patients. The analysis results for OS, DSS, DFI, and PFI showed that SLC1A5 is closely related to the prognosis of cancer patients and is a risk factor for most cancer types. We found that high expression of SLC1A5 in KIRC, MESO, LIHC, and LGG patients indicated a poor prognosis. Previous studies also suggested that SLC1A5 overexpression is significantly associated with poor OS in more than half of cancer types, including LGG and GBM [35]. It is well known that differentially expressed genes can play distinct roles in biological functions and related biological processes, thereby contributing to the divergent biological behaviors of cancer cells [45]. Building upon the evidence of the prognostic significance of SLC1A5 in gliomas, we further investigated its functional implications through GSEA.

Existing studies have indicated that IL-2 can stimulate the activation of NK cells and enhance their ability to produce INF-γ by upregulating SLC1A5 [46]. Our GSEA results showed that SLC1A5 was involved in immune activation processes, such as TNFA signaling via NFKB, IFN-α response, IFN-γ response, and inflammatory response, but this correlation was quite different in different cancer types. For example, these processes were significantly enriched in GBM, LGG, OV, PCPG, SARC, THCA, and UVM, while the results were opposite in COAD, ESCA, LUAD, and READ. This suggests that SLC1A5 plays different roles in different cancer types. A study also suggested that immune-related pathways, including TNFA signaling via NFKB, IFN-α response, and IFN-γ response, are enriched in glioma and highly correlated with tumor progression [47], which is strong supporting evidence for our results. However, there is limited research on the immunomodulatory role of SLC1A5 in glioblastoma. Therefore, we conducted a comprehensive investigation to further explore the relationship between SLC1A5 and immune regulation in glioblastoma.

Increasing evidence suggests that the proportion of immune-infiltrating cells is closely associated with the antitumor response status [8, 12, 24, 39]. Recently, certain types of infiltrating immune cells and checkpoints have been shown to alter the efficacy of immunotherapy and impact the prognosis of cancer patients [15, 48, 49]. Using the TIMER online database, we discovered a significant correlation between SLC1A5 expression and immune cell infiltration in glioma. Specifically, SLC1A5 expression was positively associated with the infiltration of CAFs, monocytes, and M2 macrophages in glioma and negatively associated with the infiltration of Tfh cells and MDSCs. Chen et al. found that CAFs do not exist as individual cells in the TME but rather interact with tumor cells, promoting tumor growth and survival and maintaining their malignant characteristics [50]. Monocytes play both protumor and antitumor roles in cancer. However, within the TME, they contribute to immune suppression, extracellular matrix (ECM) remodeling, angiogenesis, and intratumoral infiltration mediated by cancer cells [51]. Another study revealed that tumor-associated macrophages constitute a significant proportion of infiltrating immune cells and contribute to the progression of glioma [38]. Therefore, the significance of tumor-infiltrating macrophages in glioma warrants further investigation.

Next, we found a close correlation between SLC1A5 and tumor-associated macrophages. Macrophages can be classified into M1 and M2 subtypes. M1 macrophages have antitumor surface properties and exhibit inhibitory effects on tumor progression, while M2-polarized macrophages promote tumor progression by secreting vascular endothelial growth factor (VEGF) and other proangiogenic factors, such as CD163 [52]. M2-polarized macrophages suppress the tumor immune inhibitory microenvironment by secreting cytokines that inhibit T cells and other immune cell types [38, 52]. Glutamine, whose uptake is mediated by SLC1A5, can ultimately be converted to α-ketoglutarate (αKG) through the action of glutaminase [53]. αKG promotes the activation of M2 macrophages through Jmjd3-dependent metabolic and epigenetic reprogramming [54]. Conversely, αKG inhibits the activation of M1 macrophages by suppressing the nuclear factor kappa B (NF-κB) pathway [54]. Thus, Chen et al. suggested that inhibiting SLC1A5 could promote the activation of M1 macrophages, thereby facilitating the implementation of immunotherapy [55]. These findings indicate that high expression of SLC1A5 not only promotes immune cell infiltration but also induces the M2 polarization of macrophages, thereby promoting tumorigenesis. Therefore, inhibiting SLC1A5 may represent a potential novel strategy to prevent macrophage polarization toward the M2 phenotype and inhibit the progression of glioblastoma.

Several studies have suggested that targeting SLC1A5 may decrease the expression of immune checkpoint genes and enhance the efficacy of immunotherapy [44, 55]. In contrast, our findings indicate that increased SLC1A5 expression is associated with the expression of various immunomodulator genes, such as CTLA4, PD-1, PD-L1, and HAVCR2. Glioblastoma, known as a “cold” tumor, is characterized by T-cell exhaustion and poor response to immune checkpoint blockade [8, 12]. According to our research, SLC1A5 expression is significantly correlated with T-cell exhaustion and the expression of immune-suppressive markers in immune cells. Therefore, we propose that SLC1A5 may induce immune suppression in glioblastoma through mechanisms involving T-cell exhaustion, upregulation of PD-L1, and the accumulation of Tregs and MDSCs.

To assess the relationship between SLC1A5 expression and the response to ICB therapy, we analyzed the association between SLC1A5 and MSI and TMB. Clinical studies have shown that high MSI or TMB may be associated with sensitivity to immune checkpoint inhibitors, and patients with these characteristics may benefit from immunotherapy [56, 57]. We found a positive correlation between SLC1A5 expression and MSI in GBM. Notably, glioblastoma, melanoma and renal cell carcinoma (RCC) patients with high SLC1A5 expression had a worse prognosis than those with low expression when anti-PD-1 therapy was applied. In conclusion, we propose that SLC1A5 may play a role in shaping the immunosuppressive tumor microenvironment and regulating the malignant progression of glioblastoma. It has the potential to serve as a predictive biomarker for immunotherapy outcomes in glioma patients.

We then used gene expression profiling to screen for novel small-molecule inhibitors that could serve as cancer treatments. Through the analysis of drugs whose sensitivity may be related to SLC1A5 expression, we found that ingenol, prostratin, and parthenolide can be used as potential small molecule drugs for cancer treatment. A previous study showed that ingenol induces immunogenic cell death of prostate cancer cells by triggering mitophagy and apoptosis, promotes the normalization of tumor blood vessels, and allows immune cells to fully infiltrate the tumor, thereby exerting antitumor effects [58]. Therefore, it can be determined that ingenol, as an emerging antitumor drug, can affect the tumor microenvironment. Myeloid-derived suppressor cells (MDSCs), as an immature innate cell population, can produce immunosuppressive factors to suppress T cells in the tumor environment. Chaib et al. found that prostratin suppressed the expansion of MDSCs and inhibited tumor growth in breast cancer [59]. Previous studies have revealed the superior anticancer activity of parthenolide, which indicates that it has the potential to become a first-line drug [60]. Lu et al. found that parthenolide inhibits the transcriptional expression of the immune checkpoint molecule PD-L1 by targeting the phosphorylation of the transcription factor STAT3, thereby inhibiting the proliferation of GBM in mice. The ability of parthenolide to reverse the immunosuppressive state of GBM also led us to hypothesize that SLC1A5 is involved. Previous studies have shown that parthenolide activates NADPH oxidase, increases ROS levels in prostate cancer cells, and inhibits antioxidants to increase oxidative stress [60]. However, SLC1A5, as a glutamine transporter, can increase ROS production when glutamine metabolism is inhibited. Although the relationship between SLC1A5 and parthenolide in the treatment of cancer has not yet been elucidated, our findings and deduced hypotheses can provide some clues for further research.

Many current studies have shown that SLC1A5 plays an oncogenic role in many cancers, such as hepatocellular carcinoma [61], lung cancer [62], breast cancer [63], and colon cancer [64]. Our results also showed that SLC1A5 knockdown inhibited the proliferation and migration of glioma cells *in vitro*. After further study, we found that SLC1A5 is a suppressor of ferroptosis in glioma, and knockdown of SLC1A5 can downregulate the expression of GPX4. The inhibitory effect of SLC1A5 on ferroptosis may also be due to excessive cellular uptake of glutamine with subsequent formation of reducing species. Under the Warburg effect, SLC1A5 transports a large amount of glutamine into the cell, and glutamine is metabolized to generate a large amount of ROS [65]. However, the high metabolic demands of tumors can cause oxidative stress-related damage and affect tumor growth [66]. Therefore, the upregulation of SLC1A5 inhibits the production of MDA and upregulates GPX4 to reduce oxidative stress-related damage, thereby accelerating cell proliferation and promoting malignant tumor progression.

Our study still has some limitations. We predicted through bioinformatics analysis that SLC1A5 is involved in regulating the tumor microenvironment in most cancer types and is closely related to immune cell infiltration, but the importance of SLC1A5 has been experimentally confirmed in only a few cancer types. In addition, we identified potential drugs related to SLC1A5, but we did not demonstrate a direct interaction between SLC1A5 and these drugs, and the underlying mechanism remains unknown. Since the analysis was based on an open public database, the data results inevitably have bias; therefore, we believe that more comprehensive and refined research on the mechanism and clinical application of SLC1A5 is still needed.

In conclusion, we conducted a pancancer analysis of SLC1A5, demonstrated its role as a prognostic biomarker in cancer patients, explored its potential biological functions, and found that it can effectively predict patient response to immunotherapy. These results indicated that therapy targeting SLC1A5 could be an effective method of cancer treatment.

## MATERIALS AND METHODS

### Data source

The mRNA expression profile and clinical characteristics of patients from the TCGA pancancer and GTEx cohorts were downloaded from the UCSC Xena database (https://xenabrowser.net/datapages/). The cBioPortal for Cancer Genomics (http://cbioportal.org) web tool was used to analyze the genomic alteration frequency of SLC1A5 in the 33 cancer types. Human Protein Atlas (THPA, https://www.proteinatlas.org/) was used to detect SLC1A5 protein expression levels in pathological tissues and to confirm protein distribution at the subcellular level. The compartmentalized protein–protein interaction database (ComPPI) (http://comppi.linkgroup.hu) was used to detect the protein‒protein interaction information. A list of the cancers and their abbreviations are presented in Supplementary Table 2.

### Single-cell analysis of SLC1A5

The Tumor Immune Single-cell Hub (TISCH) web tool was used to analyze gene expression profiles in tumor microenvironment cells at the single-cell level. Analysis parameters included genes, major lineages, and all cancers selected in the online tool. SLC1A5 expression levels in each cell type were quantified and visualized by a heatmap. Documentation of the data collection, processing and analysis procedures is available online (http://tisch.comp-genomics.org/documentation/).

### Pancancer prognosis analysis of SLC1A5

Four key outcome measures were obtained from the UCSC Xena database (https://xenabrowser.net/datapages/), including overall survival (OS), disease-specific survival (DSS), disease-free interval (DFI), and progression-free interval (PFS). Log-rank and univariate Cox regression tests were used to evaluate the association between the expression of SLC1A5 and the prognosis of the patients in each type of cancer. In univariate Cox regression models, SLC1A5 expression was treated as a continuous variable, and Wald’s test was used to assess significant differences in survival. Kaplan‒Meier (KM) curves were used to assess survival and compared using log-rank tests. Risk ratios (HRs) with 95% confidence intervals (95% CIs) were calculated for log-rank and univariate Cox regression, with HR > 1 indicating a risk factor and HR < 1 indicating a protective factor, and the results are presented as a heatmap.

### Genes with differential expression between the low- and high-SLC1A5 subgroups

Patients were ranked by SLC1A5 expression level, with the top 30% defined as the high SLC1A5 group and the bottom 30% defined as the low SLC1A5 group. The log2 (fold change) and adjusted *p* value of each gene in each tumor were calculated using the limma package to determine the differentially expressed genes (DEGs) between the high and low SLC1A5 groups (Supplementary Table 3). Genes with *p*-adjusted values < 0.05 were considered DEGs.

### Gene set enrichment analysis

For enrichment analysis, the normalized enrichment score (NES) and false discovery rate (FDR) of each biological process in each cancer type were calculated by downloading the “gmt” file of the hallmark gene set (h.all.v7.4.symbols.gmt) from the GSEA website (https://www.gsea-msigdb.org/gsea/index.jsp). GSEA was implemented using the clusterProfiler R package, and the R package ggplot2 was used to generate the bubble plot.

### Immune cell infiltration analysis in TIMER2

Tumor Immune Estimation Resource (TIMER) is a comprehensive resource for the systematic analysis of immune infiltrates across different types of cancer. For all cancers in TCGA, immune cell infiltration data were downloaded from TIMER2.0 (http://timer.cistrome.org). To investigate the immune infiltration data, we identified the correlations between the expression of SLC1A5 mRNA and 21 immune cells, including B cells, CD4+ T cells, CD8+ T cells, dendritic cells, endothelial cells (Endo), eosinophils (Eos), cancer-associated fibroblasts (CAFs), lymphoid progenitors, myeloid progenitors, monocyte progenitors, mast cells, macrophages, monocytes, neutrophils, hematopoietic stem cells (HSCs), NK cells, T-cell follicular helper cells, γ/δ T cells, NK T cells and regulatory T cells (Tregs), in pancancer by Spearman correlation analysis.

### Immunotherapy prediction analysis

A Spearman correlation test was used in this study to determine the correlation between SLC1A5 expression levels and levels of immunomodulators, including 46 immunostimulators and 24 immunoinhibitors with information downloaded from the TISIDB database (http://cis.hku.hk/TISIDB/download.php). Furthermore, we investigated the relationship between SLC1A5 expression and tumor mutation burden (TMB) and microsatellite instability (MSI) across cancers. We performed visual analysis of the immunotherapy response and survival of SLC1A5 in different cancers using the Immunotherapy Response module in the TIGER (Tumor Immunotherapy Gene Expression Resource) online database (http://tiger.canceromics.org/).

### Compounds correlating with SLC1A5 in pancancer

The Connectivity Map (CMap) is a gene expression profiling database based on drug-related gene expression developed by the Broad Institute. It is mainly used to reveal the functional relationship between small molecule compounds, genes and disease states. We analyzed the correlation of SLC1A5 expression levels and the levels of genes related to small molecule inhibitors in each cancer by CMap based on DEGs between the low-SLC1A5 and high-SLC1A5 subgroups. In a previous report, the steps for applying the web tool and processing the data for heatmap visualization were described in detail [67].

### Cell culture, small interfering RNA transfection, RT‒qPCR, and Western blotting

NHA, T98, U118, LN229, U251, and U87 cell lines were obtained from the Cancer Cell Bank of the Chinese Academy of Medical Sciences (Beijing, China). The cells were cultured as described in our previous studies [68, 69]. Small interfering RNA (siRNA) targeting SLC1A5 was synthesized by General Biologicals (AnHui, China). The RNA Isolater Total RNA Extraction Reagent (BioFlux, HangZhou, China) was used for RNA extraction. The reverse transcription and real-time PCR procedures were performed as previously described [68, 69]. The following antibodies were used for the Western blot (WB) assay: anti-Actin antibody (Abcam, 1:1000), anti-SLC1A5 antibody, anti-GPX4 antibody, anti-ACSL4 antibody, and anti-TFRC antibody. Western blot assays were performed as described in our previous study [68, 69].

### Cell proliferation analyses, colony formation assay and migration assay

For the *in vitro* cell proliferation assay, the CCK-8 assay was performed using the CCK-8 kit (Bioss, Beijing, China) according to the manufacturer’s protocol. An enzyme immunoassay instrument (Thermo Fisher Scientific, USA) was used to detect the OD value at 450 nm. An *in vitro* colony formation assay was performed to determine whether colony formation was possible *in vitro*, and the procedure was performed as previously described [68, 69]. Finally, the migration ability of the cells was determined using the Transwell chamber system (BD Biosciences, BD Biosciences, Franklin Lakes, NJ, USA).

### Assay for GSH/GSSG and MDA

The cells were harvested, and the intracellular GSH/GSSG ratio was measured spectrophotometrically using a GSH/GSSG assay kit (Beyotime Biotechnology, Shanghai, China) according to the kit instructions. The absorbance at 593 nm was measured using a microplate reader (Bio-Rad, Hercules, CA, USA) for colorimetric analysis. A lipid peroxidation (MDA) assay kit (Beyotime Biotechnology, Shanghai, China) was used to measure the lipid peroxidation product malondialdehyde (MDA). The operational steps of the MDA assay were performed strictly according to the instructions of the kit. The absorbance of the supernatant was measured at 532 nm using a microplate reader.

### Statistical analysis

To determine the significance of differences in expression levels between normal and tumor tissues, the Wilcoxon rank sum test was performed. The prognostic effect of SLC1A5 in each cancer was evaluated using the Kaplan-Meier method and univariate Cox proportional hazards regression analysis. For correlation analyses of SLC1A5 and other factors, a Spearman correlation analysis was performed. All the R packages were run through R Studio version 1.3.959, while all the statistical analyses were conducted using R version 4.2.2 (https://www.r-project.org/) and Prism 8 (GraphPad Software Inc., La Jolla, CA, USA).

## Supplementary Materials

Supplementary Figures

Supplementary Table 1

Supplementary Table 2

Supplementary Table 3

## References

[1] Deo SVS, Sharma J, Kumar S. GLOBOCAN 2020 Report on Global Cancer Burden: Challenges and Opportunities for Surgical Oncologists. Ann Surg Oncol. 2022; 29:6497–500. 10.1245/s10434-022-12151-6 35838905

[2] Siegel RL, Miller KD, Fuchs HE, Jemal A. Cancer statistics, 2022. CA Cancer J Clin. 2022; 72:7–33. 10.3322/caac.21708 35020204

[3] Torp SH, Solheim O, Skjulsvik AJ. The WHO 2021 Classification of Central Nervous System tumours: a practical update on what neurosurgeons need to know-a minireview. Acta Neurochir (Wien). 2022; 164:2453–64. 10.1007/s00701-022-05301-y 35879477 PMC9427889

[4] Ostrom QT, Cioffi G, Waite K, Kruchko C, Barnholtz-Sloan JS. CBTRUS Statistical Report: Primary Brain and Other Central Nervous System Tumors Diagnosed in the United States in 2014-2018. Neuro Oncol. 2021; 23:iii1–105. 10.1093/neuonc/noab200 34608945 PMC8491279

[5] Hambardzumyan D, Bergers G. Glioblastoma: Defining Tumor Niches. Trends Cancer. 2015; 1:252–65. 10.1016/j.trecan.2015.10.009 27088132 PMC4831073

[6] Tan AC, Ashley DM, López GY, Malinzak M, Friedman HS, Khasraw M. Management of glioblastoma: State of the art and future directions. CA Cancer J Clin. 2020; 70:299–312. 10.3322/caac.21613 32478924

[7] Bagley SJ, Kothari S, Rahman R, Lee EQ, Dunn GP, Galanis E, Chang SM, Nabors LB, Ahluwalia MS, Stupp R, Mehta MP, Reardon DA, Grossman SA, et al. Glioblastoma Clinical Trials: Current Landscape and Opportunities for Improvement. Clin Cancer Res. 2022; 28:594–602. 10.1158/1078-0432.CCR-21-2750 34561269 PMC9044253

[8] Yu MW, Quail DF. Immunotherapy for Glioblastoma: Current Progress and Challenges. Front Immunol. 2021; 12:676301. 10.3389/fimmu.2021.676301 34054867 PMC8158294

[9] Lichty BD, Breitbach CJ, Stojdl DF, Bell JC. Going viral with cancer immunotherapy. Nat Rev Cancer. 2014; 14:559–67. 10.1038/nrc3770 24990523

[10] Arner EN, Rathmell JC. Metabolic programming and immune suppression in the tumor microenvironment. Cancer Cell. 2023; 41:421–33. 10.1016/j.ccell.2023.01.009 36801000 PMC10023409

[11] DePeaux K, Delgoffe GM. Metabolic barriers to cancer immunotherapy. Nat Rev Immunol. 2021; 21:785–97. 10.1038/s41577-021-00541-y 33927375 PMC8553800

[12] Lim M, Xia Y, Bettegowda C, Weller M. Current state of immunotherapy for glioblastoma. Nat Rev Clin Oncol. 2018; 15:422–42. 10.1038/s41571-018-0003-5 29643471

[13] Desjardins A, Gromeier M, Herndon JE 2nd, Beaubier N, Bolognesi DP, Friedman AH, Friedman HS, McSherry F, Muscat AM, Nair S, Peters KB, Randazzo D, Sampson JH, et al. Recurrent Glioblastoma Treated with Recombinant Poliovirus. N Engl J Med. 2018; 379:150–61. 10.1056/NEJMoa1716435 29943666 PMC6065102

[14] Weller M, Kaulich K, Hentschel B, Felsberg J, Gramatzki D, Pietsch T, Simon M, Westphal M, Schackert G, Tonn JC, von Deimling A, Davis T, Weiss WA, et al, and German Glioma Network. Assessment and prognostic significance of the epidermal growth factor receptor vIII mutation in glioblastoma patients treated with concurrent and adjuvant temozolomide radiochemotherapy. Int J Cancer. 2014; 134:2437–47. 10.1002/ijc.28576 24614983

[15] Liau LM, Ashkan K, Tran DD, Campian JL, Trusheim JE, Cobbs CS, Heth JA, Salacz M, Taylor S, D'Andre SD, Iwamoto FM, Dropcho EJ, Moshel YA, et al. First results on survival from a large Phase 3 clinical trial of an autologous dendritic cell vaccine in newly diagnosed glioblastoma. J Transl Med. 2018; 16:142. 10.1186/s12967-018-1507-6 29843811 PMC5975654

[16] Reardon DA, Desjardins A, Vredenburgh JJ, O'Rourke DM, Tran DD, Fink KL, Nabors LB, Li G, Bota DA, Lukas RV, Ashby LS, Duic JP, Mrugala MM, et al, and ReACT trial investigators. Rindopepimut with Bevacizumab for Patients with Relapsed EGFRvIII-Expressing Glioblastoma (ReACT): Results of a Double-Blind Randomized Phase II Trial. Clin Cancer Res. 2020; 26:1586–94. 10.1158/1078-0432.CCR-18-1140 32034072

[17] Marcus L, Lemery SJ, Keegan P, Pazdur R. FDA Approval Summary: Pembrolizumab for the Treatment of Microsatellite Instability-High Solid Tumors. Clin Cancer Res. 2019; 25:3753–8. 10.1158/1078-0432.CCR-18-4070 30787022

[18] Amatangelo MD, Bassi DE, Klein-Szanto AJ, Cukierman E. Stroma-derived three-dimensional matrices are necessary and sufficient to promote desmoplastic differentiation of normal fibroblasts. Am J Pathol. 2005; 167:475–88. 10.1016/S0002-9440(10)62991-4 16049333 PMC1603576

[19] Sullivan MR, Danai LV, Lewis CA, Chan SH, Gui DY, Kunchok T, Dennstedt EA, Vander Heiden MG, Muir A. Quantification of microenvironmental metabolites in murine cancers reveals determinants of tumor nutrient availability. Elife. 2019; 8:e44235. 10.7554/eLife.44235 30990168 PMC6510537

[20] Reinfeld BI, Madden MZ, Wolf MM, Chytil A, Bader JE, Patterson AR, Sugiura A, Cohen AS, Ali A, Do BT, Muir A, Lewis CA, Hongo RA, et al. Cell-programmed nutrient partitioning in the tumour microenvironment. Nature. 2021; 593:282–8. 10.1038/s41586-021-03442-1 33828302 PMC8122068

[21] Hyroššová P, Milošević M, Škoda J, Vachtenheim J Jr, Rohlena J, Rohlenová K. Effects of metabolic cancer therapy on tumor microenvironment. Front Oncol. 2022; 12:1046630. 10.3389/fonc.2022.1046630 36582801 PMC9793001

[22] Li X, Sun X, Carmeliet P. Hallmarks of Endothelial Cell Metabolism in Health and Disease. Cell Metab. 2019; 30:414–33. 10.1016/j.cmet.2019.08.011 31484054

[23] Ryan DG, O'Neill LAJ. Krebs Cycle Reborn in Macrophage Immunometabolism. Annu Rev Immunol. 2020; 38:289–313. 10.1146/annurev-immunol-081619-104850 31986069

[24] Makowski L, Chaib M, Rathmell JC. Immunometabolism: From basic mechanisms to translation. Immunol Rev. 2020; 295:5–14. 10.1111/imr.12858 32320073 PMC8056251

[25] Klein Geltink RI, O'Sullivan D, Corrado M, Bremser A, Buck MD, Buescher JM, Firat E, Zhu X, Niedermann G, Caputa G, Kelly B, Warthorst U, Rensing-Ehl A, et al. Mitochondrial Priming by CD28. Cell. 2017; 171:385–97.e11. 10.1016/j.cell.2017.08.018 28919076 PMC5637396

[26] Parry RV, Chemnitz JM, Frauwirth KA, Lanfranco AR, Braunstein I, Kobayashi SV, Linsley PS, Thompson CB, Riley JL. CTLA-4 and PD-1 receptors inhibit T-cell activation by distinct mechanisms. Mol Cell Biol. 2005; 25:9543–53. 10.1128/MCB.25.21.9543-9553.2005 16227604 PMC1265804

[27] Siska PJ, Beckermann KE, Mason FM, Andrejeva G, Greenplate AR, Sendor AB, Chiang YJ, Corona AL, Gemta LF, Vincent BG, Wang RC, Kim B, Hong J, et al. Mitochondrial dysregulation and glycolytic insufficiency functionally impair CD8 T cells infiltrating human renal cell carcinoma. JCI Insight. 2017; 2:93411. 10.1172/jci.insight.93411 28614802 PMC5470888

[28] Zandberg DP, Menk AV, Velez M, Normolle D, DePeaux K, Liu A, Ferris RL, Delgoffe GM. Tumor hypoxia is associated with resistance to PD-1 blockade in squamous cell carcinoma of the head and neck. J Immunother Cancer. 2021; 9:e002088. 10.1136/jitc-2020-002088 33986123 PMC8126285

[29] Song W, Li D, Tao L, Luo Q, Chen L. Solute carrier transporters: the metabolic gatekeepers of immune cells. Acta Pharm Sin B. 2020; 10:61–78. 10.1016/j.apsb.2019.12.006 31993307 PMC6977534

[30] Cormerais Y, Massard PA, Vucetic M, Giuliano S, Tambutté E, Durivault J, Vial V, Endou H, Wempe MF, Parks SK, Pouyssegur J. The glutamine transporter ASCT2 (SLC1A5) promotes tumor growth independently of the amino acid transporter LAT1 (SLC7A5). J Biol Chem. 2018; 293:2877–87. 10.1074/jbc.RA117.001342 29326164 PMC5827425

[31] Kekuda R, Prasad PD, Fei YJ, Torres-Zamorano V, Sinha S, Yang-Feng TL, Leibach FH, Ganapathy V. Cloning of the sodium-dependent, broad-scope, neutral amino acid transporter Bo from a human placental choriocarcinoma cell line. J Biol Chem. 1996; 271:18657–61. 10.1074/jbc.271.31.18657 8702519

[32] Bode BP. Recent molecular advances in mammalian glutamine transport. J Nutr. 2001; 131:2475S. 10.1093/jn/131.9.2475S 11533296

[33] El-Ansari R, Craze ML, Alfarsi L, Soria D, Diez-Rodriguez M, Nolan CC, Ellis IO, Rakha EA, Green AR. The combined expression of solute carriers is associated with a poor prognosis in highly proliferative ER+ breast cancer. Breast Cancer Res Treat. 2019; 175:27–38. 10.1007/s10549-018-05111-w 30671766

[34] Liu X, Qin H, Li Z, Lv Y, Feng S, Zhuang W, Quan X, Guo C, Chen C, Zhang H. Inspiratory hyperoxia suppresses lung cancer metastasis through a MYC/SLC1A5-dependent metabolic pathway. Eur Respir J. 2022; 60:2200062. 10.1183/13993003.00062-2022 35680143 PMC9712851

[35] Zhang H, Cui K, Yao S, Yin Y, Liu D, Huang Z. Comprehensive molecular and clinical characterization of SLC1A5 in human cancers. Pathol Res Pract. 2021; 224:153525. 10.1016/j.prp.2021.153525 34171602

[36] Syafruddin SE, Nazarie WFW, Moidu NA, Soon BH, Mohtar MA. Integration of RNA-Seq and proteomics data identifies glioblastoma multiforme surfaceome signature. BMC Cancer. 2021; 21:850. 10.1186/s12885-021-08591-0 34301218 PMC8306276

[37] Schulte ML, Fu A, Zhao P, Li J, Geng L, Smith ST, Kondo J, Coffey RJ, Johnson MO, Rathmell JC, Sharick JT, Skala MC, Smith JA, et al. Pharmacological blockade of ASCT2-dependent glutamine transport leads to antitumor efficacy in preclinical models. Nat Med. 2018; 24:194–202. 10.1038/nm.4464 29334372 PMC5803339

[38] Akkari L, Bowman RL, Tessier J, Klemm F, Handgraaf SM, de Groot M, Quail DF, Tillard L, Gadiot J, Huse JT, Brandsma D, Westerga J, Watts C, Joyce JA. Dynamic changes in glioma macrophage populations after radiotherapy reveal CSF-1R inhibition as a strategy to overcome resistance. Sci Transl Med. 2020; 12:eaaw7843. 10.1126/scitranslmed.aaw7843 32669424

[39] Shiravand Y, Khodadadi F, Kashani SMA, Hosseini-Fard SR, Hosseini S, Sadeghirad H, Ladwa R, O'Byrne K, Kulasinghe A. Immune Checkpoint Inhibitors in Cancer Therapy. Curr Oncol. 2022; 29:3044–60. 10.3390/curroncol29050247 35621637 PMC9139602

[40] Hsieh YY, Cheng YW, Wei PL, Yang PM. Repurposing of ingenol mebutate for treating human colorectal cancer by targeting S100 calcium-binding protein A4 (S100A4). Toxicol Appl Pharmacol. 2022; 449:116134. 10.1016/j.taap.2022.116134 35724704

[41] Alotaibi D, Amara S, Johnson TL, Tiriveedhi V. Potential anticancer effect of prostratin through SIK3 inhibition. Oncol Lett. 2018; 15:3252–8. 10.3892/ol.2017.7674 29435066 PMC5778866

[42] Ghahremanloo A, Soltani A, Modaresi SMS, Hashemy SI. Recent advances in the clinical development of immune checkpoint blockade therapy. Cell Oncol (Dordr). 2019; 42:609–26. 10.1007/s13402-019-00456-w 31201647 PMC12994343

[43] Garattini S, Fuso Nerini I, D'Incalci M. Not only tumor but also therapy heterogeneity. Ann Oncol. 2018; 29:13–9. 10.1093/annonc/mdx646 29045538

[44] Huang F, Zhao Y, Zhao J, Wu S, Jiang Y, Ma H, Zhang T. Upregulated SLC1A5 promotes cell growth and survival in colorectal cancer. Int J Clin Exp Pathol. 2014; 7:6006–14. 25337245 PMC4203216

[45] Saliani M, Jalal R, Javadmanesh A. Differential expression analysis of genes and long non-coding RNAs associated with KRAS mutation in colorectal cancer cells. Sci Rep. 2022; 12:7965. 10.1038/s41598-022-11697-5 35562390 PMC9106686

[46] Jensen H, Potempa M, Gotthardt D, Lanier LL. Cutting Edge: IL-2-Induced Expression of the Amino Acid Transporters SLC1A5 and CD98 Is a Prerequisite for NKG2D-Mediated Activation of Human NK Cells. J Immunol. 2017; 199:1967–72. 10.4049/jimmunol.1700497 28784848 PMC5587401

[47] Gieryng A, Pszczolkowska D, Walentynowicz KA, Rajan WD, Kaminska B. Immune microenvironment of gliomas. Lab Invest. 2017; 97:498–518. 10.1038/labinvest.2017.19 28287634

[48] de Streel G, Lucas S. Targeting immunosuppression by TGF-β1 for cancer immunotherapy. Biochem Pharmacol. 2021; 192:114697. 10.1016/j.bcp.2021.114697 34302795 PMC8484859

[49] Weller M, Butowski N, Tran DD, Recht LD, Lim M, Hirte H, Ashby L, Mechtler L, Goldlust SA, Iwamoto F, Drappatz J, O'Rourke DM, Wong M, et al, and ACT IV trial investigators. Rindopepimut with temozolomide for patients with newly diagnosed, EGFRvIII-expressing glioblastoma (ACT IV): a randomised, double-blind, international phase 3 trial. Lancet Oncol. 2017; 18:1373–85. 10.1016/S1470-2045(17)30517-X 28844499

[50] Chen Y, McAndrews KM, Kalluri R. Clinical and therapeutic relevance of cancer-associated fibroblasts. Nat Rev Clin Oncol. 2021; 18:792–804. 10.1038/s41571-021-00546-5 34489603 PMC8791784

[51] de Visser KE, Joyce JA. The evolving tumor microenvironment: From cancer initiation to metastatic outgrowth. Cancer Cell. 2023; 41:374–403. 10.1016/j.ccell.2023.02.016 36917948

[52] Xu Y, Wang X, Liu L, Wang J, Wu J, Sun C. Role of macrophages in tumor progression and therapy (Review). Int J Oncol. 2022; 60:57. 10.3892/ijo.2022.5347 35362544 PMC8997338

[53] Stunault MI, Bories G, Guinamard RR, Ivanov S. Metabolism Plays a Key Role during Macrophage Activation. Mediators Inflamm. 2018; 2018:2426138. 10.1155/2018/2426138 30647530 PMC6311794

[54] Liu PS, Wang H, Li X, Chao T, Teav T, Christen S, Di Conza G, Cheng WC, Chou CH, Vavakova M, Muret C, Debackere K, Mazzone M, et al. α-ketoglutarate orchestrates macrophage activation through metabolic and epigenetic reprogramming. Nat Immunol. 2017; 18:985–94. 10.1038/ni.3796 28714978

[55] Chen R, Chen L. Solute carrier transporters: emerging central players in tumour immunotherapy. Trends Cell Biol. 2022; 32:186–201. 10.1016/j.tcb.2021.08.002 34511324

[56] Jardim DL, Goodman A, de Melo Gagliato D, Kurzrock R. The Challenges of Tumor Mutational Burden as an Immunotherapy Biomarker. Cancer Cell. 2021; 39:154–73. 10.1016/j.ccell.2020.10.001 33125859 PMC7878292

[57] Goodman AM, Sokol ES, Frampton GM, Lippman SM, Kurzrock R. Microsatellite-Stable Tumors with High Mutational Burden Benefit from Immunotherapy. Cancer Immunol Res. 2019; 7:1570–3. 10.1158/2326-6066.CIR-19-0149 31405947 PMC6774837

[58] Wang Z, Sun C, Wu H, Xie J, Zhang T, Li Y, Xu X, Wang P, Wang C. Cascade targeting codelivery of ingenol-3-angelate and doxorubicin for enhancing cancer chemoimmunotherapy through synergistic effects in prostate cancer. Mater Today Bio. 2021; 13:100189. 10.1016/j.mtbio.2021.100189 34977528 PMC8686035

[59] Chaib M, Sipe LM, Yarbro JR, Bohm MS, Counts BR, Tanveer U, Pingili AK, Daria D, Marion TN, Carson JA, Thomas PG, Makowski L. PKC agonism restricts innate immune suppression, promotes antigen cross-presentation and synergizes with agonistic CD40 antibody therapy to activate CD8^+^ T cells in breast cancer. Cancer Lett. 2022; 531:98–108. 10.1016/j.canlet.2022.01.017 35074498 PMC9867936

[60] Liu X, Wang X. Recent advances on the structural modification of parthenolide and its derivatives as anticancer agents. Chin J Nat Med. 2022; 20:814–29. 10.1016/S1875-5364(22)60238-3 36427916

[61] Bothwell PJ, Kron CD, Wittke EF, Czerniak BN, Bode BP. Targeted Suppression and Knockout of ASCT2 or LAT1 in Epithelial and Mesenchymal Human Liver Cancer Cells Fail to Inhibit Growth. Int J Mol Sci. 2018; 19:2093. 10.3390/ijms19072093 30029480 PMC6073291

[62] Hassanein M, Hoeksema MD, Shiota M, Qian J, Harris BK, Chen H, Clark JE, Alborn WE, Eisenberg R, Massion PP. SLC1A5 mediates glutamine transport required for lung cancer cell growth and survival. Clin Cancer Res. 2013; 19:560–70. 10.1158/1078-0432.CCR-12-2334 23213057 PMC3697078

[63] van Geldermalsen M, Wang Q, Nagarajah R, Marshall AD, Thoeng A, Gao D, Ritchie W, Feng Y, Bailey CG, Deng N, Harvey K, Beith JM, Selinger CI, et al. ASCT2/SLC1A5 controls glutamine uptake and tumour growth in triple-negative basal-like breast cancer. Oncogene. 2016; 35:3201–8. 10.1038/onc.2015.381 26455325 PMC4914826

[64] He W, Tao W, Zhang F, Jie Q, He Y, Zhu W, Tan J, Shen W, Li L, Yang Y, Cheng H, Sun D. Lobetyolin induces apoptosis of colon cancer cells by inhibiting glutamine metabolism. J Cell Mol Med. 2020; 24:3359–69. 10.1111/jcmm.15009 31990147 PMC7131919

[65] Gao M, Monian P, Quadri N, Ramasamy R, Jiang X. Glutaminolysis and Transferrin Regulate Ferroptosis. Mol Cell. 2015; 59:298–308. 10.1016/j.molcel.2015.06.011 26166707 PMC4506736

[66] Kim SJ, Kim HS, Seo YR. Understanding of ROS-Inducing Strategy in Anticancer Therapy. Oxid Med Cell Longev. 2019; 2019:5381692. 10.1155/2019/5381692 31929855 PMC6939418

[67] Malta TM, Sokolov A, Gentles AJ, Burzykowski T, Poisson L, Weinstein JN, Kamińska B, Huelsken J, Omberg L, Gevaert O, Colaprico A, Czerwińska P, Mazurek S, et al, and Cancer Genome Atlas Research Network. Machine Learning Identifies Stemness Features Associated with Oncogenic Dedifferentiation. Cell. 2018; 173:338–54.e15. 10.1016/j.cell.2018.03.034 29625051 PMC5902191

[68] Gui S, Chen P, Liu Y, Chen Q, Cheng T, Lv S, Zhou T, Song Z, Xiao J, He W, Yuan S, Cheng Z. TUBA1C expression promotes proliferation by regulating the cell cycle and indicates poor prognosis in glioma. Biochem Biophys Res Commun. 2021; 577:130–8. 10.1016/j.bbrc.2021.08.079 34517210

[69] Wu Y, Du H, Zhan M, Wang H, Chen P, Du D, Liu X, Huang X, Ma P, Peng D, Sun L, Yuan S, Ding J, et al. Sepiapterin reductase promotes hepatocellular carcinoma progression via FoxO3a/Bim signaling in a nonenzymatic manner. Cell Death Dis. 2020; 11:248. 10.1038/s41419-020-2471-7 32312975 PMC7170898

