# Supplementary material for: SLC1A5 is a novel biomarker associated with ferroptosis and the tumor microenvironment: a pancancer analysis
Source: Aging (Albany NY). 2023 Aug 10;15(15):7451–75. doi: 10.18632/aging.204911 (PMC10457057; doi:10.18632/aging.204911)
Supplement: Supplementary Figures [file aging-15-204911-s001.pdf]

SUPPLEMENTARY FIGURES

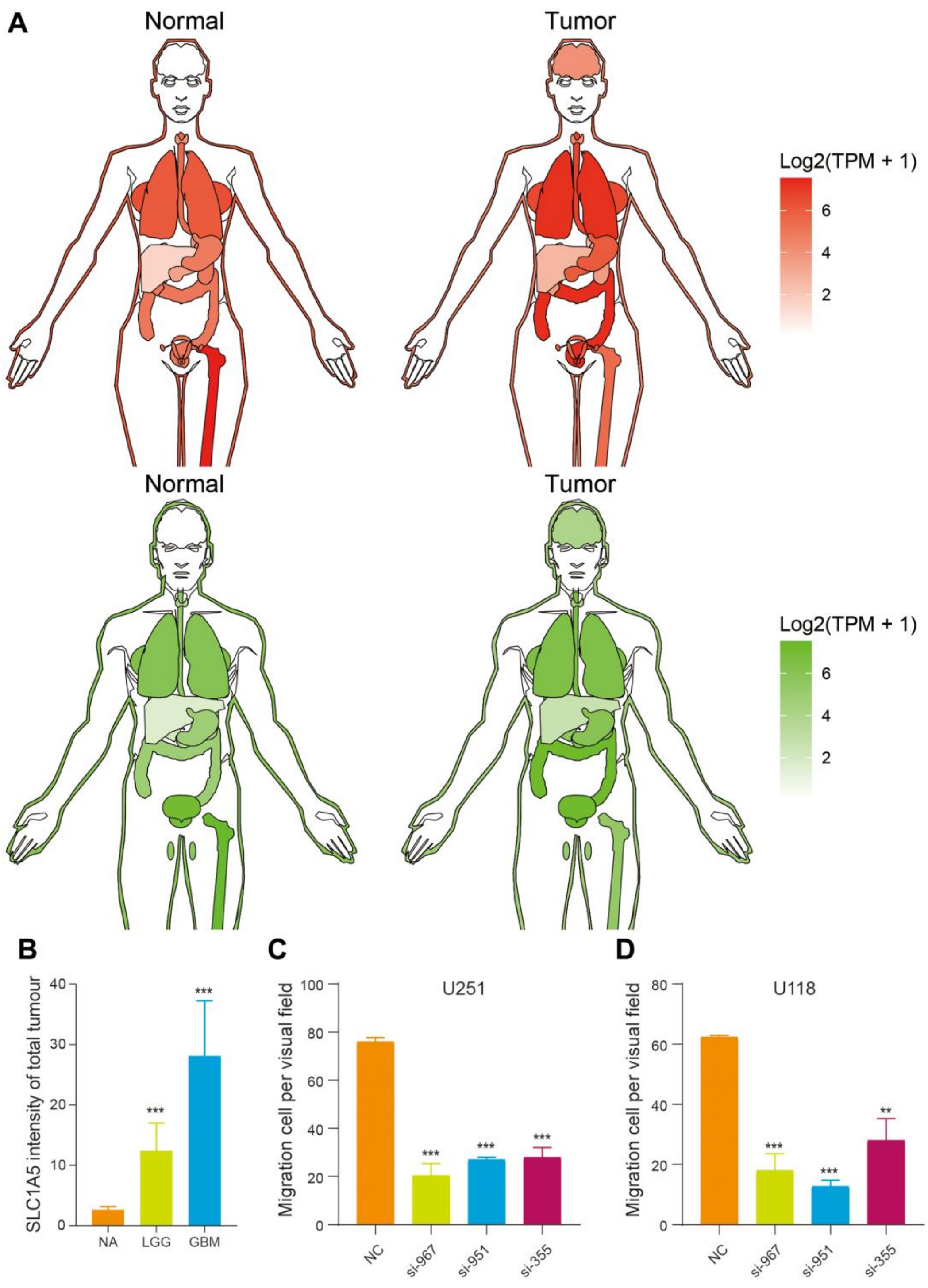

Supplementary Figure 1. (**A**) The expression distribution of SLC1A5 in normal organs and tumor tissues of men and women is shown in the anatomical diagram of human organs. (**B**) Statistical graph of immunohistochemical staining results of SLC1A5 expression levels in glioma tissue and normal brain tissue. (**C**, **D**) Statistical results of transwell migration experiments following SLC1A5 knockdown in U251 and U118 cell lines. The labeled asterisk indicates the statistical *p* value ($^{*}p < 0.05$, $^{**}p < 0.01$, $^{***}p < 0.001$).

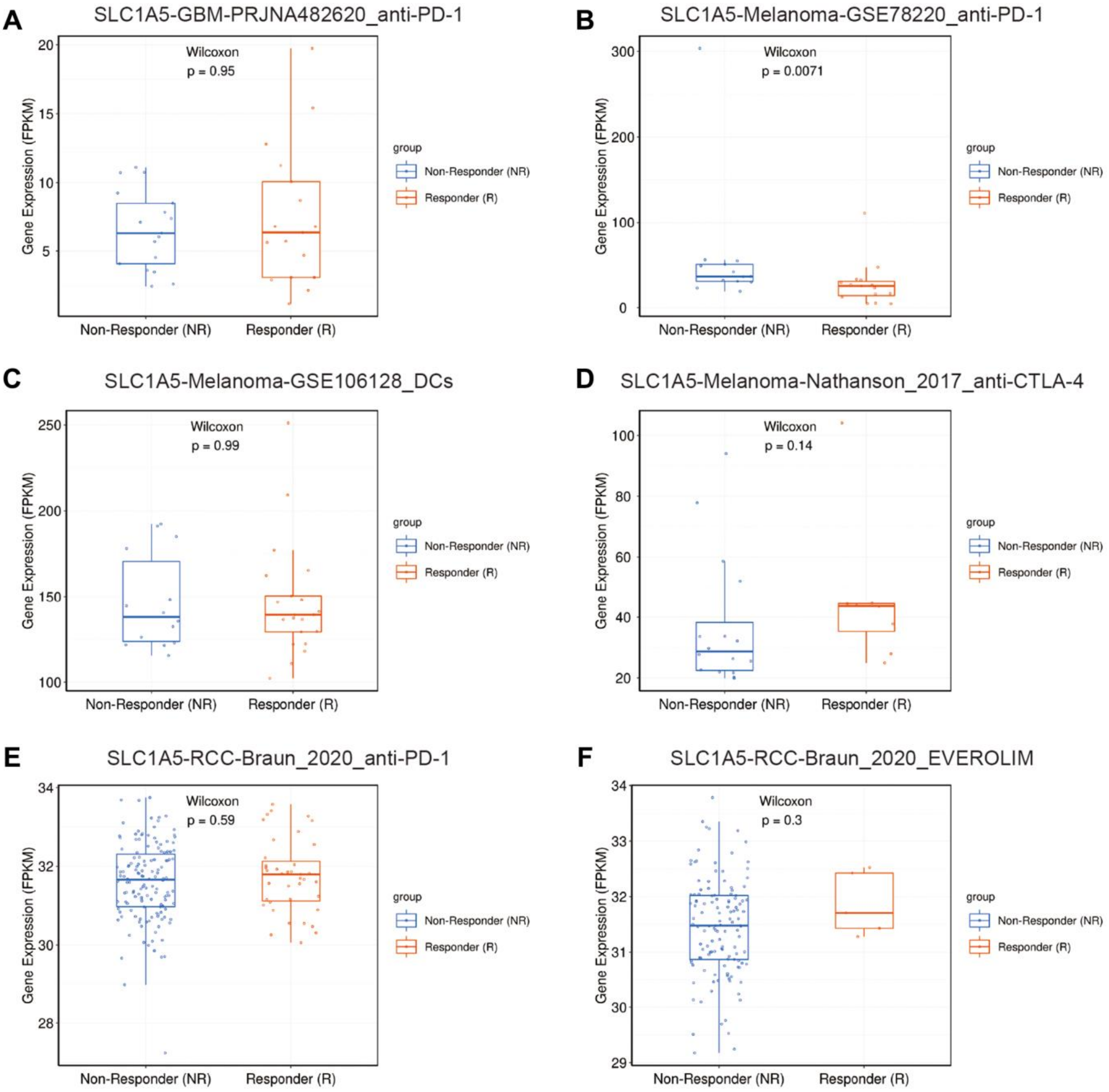

Supplementary Figure 2. Differential expression between responders and nonresponders. (**A**) The expression levels of SLC1A5 in responders and nonresponders among GBM patients following ai-PD-1 treatment in the PRJNA482620 cohort. (**B**) The expression levels of SLC1A5 in responders and nonresponders among melanoma patients following ai-PD-1 treatment in the GSE78220 cohort. (**C**) The expression levels of SLC1A5 in responders and nonresponders among melanoma patients following DC treatment in the GSE106128 cohort. (**D**) The expression levels of SLC1A5 in responders and nonresponders among melanoma patients following anti-CTLA4 treatment in the Nathanson_2017 cohort. (**E**) The expression levels of SLC1A5 in responders and nonresponders among renal cell carcinoma patients following anti-PD-1 treatment in the Braun_2020 cohort. (**F**) The expression levels of SLC1A5 in responders and nonresponders among renal cell carcinoma patients following EVEROLIM treatment in the Braun_2020 cohort.

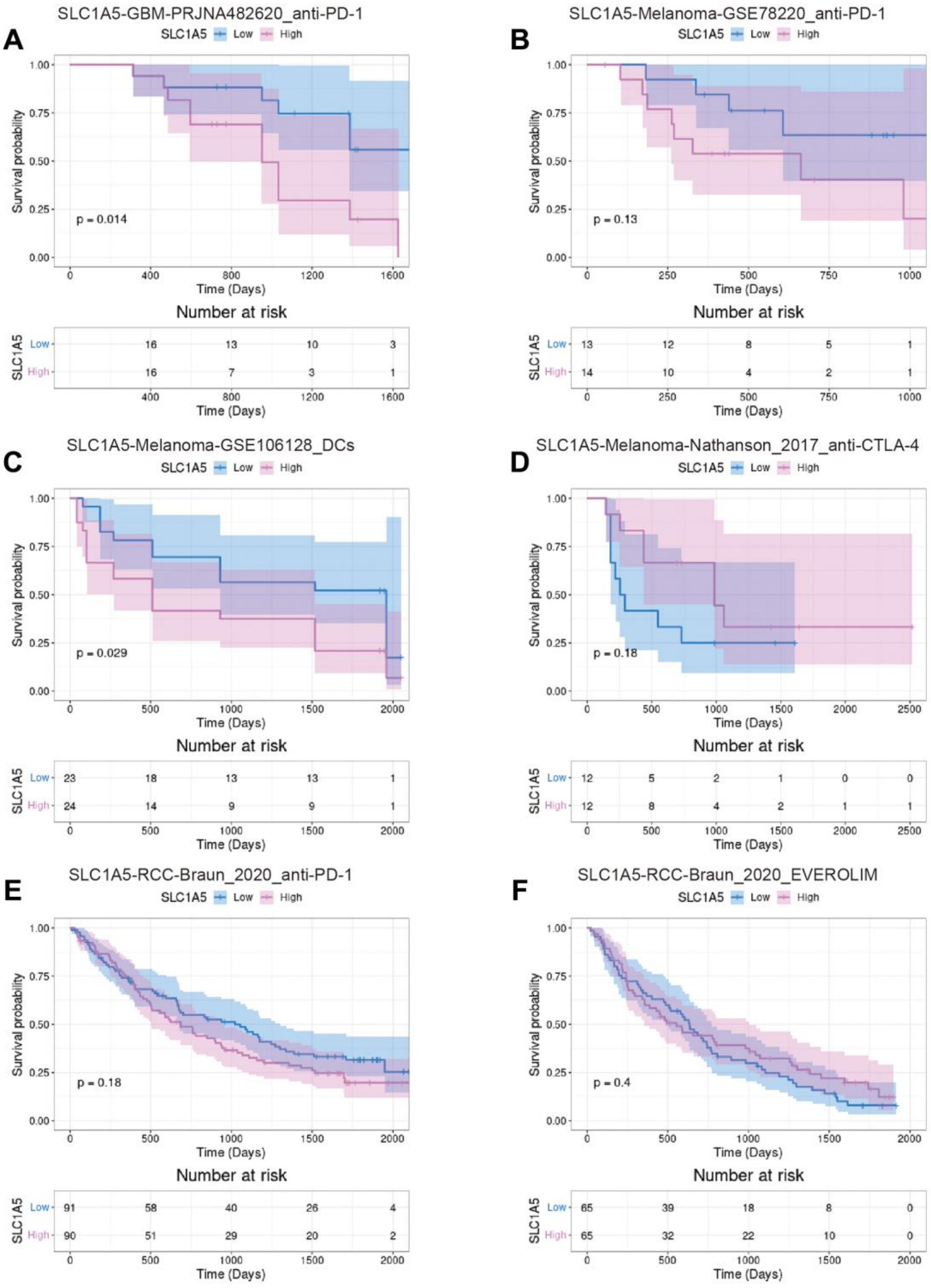

Supplementary Figure 3. Survival analysis of patients after immune checkpoint inhibitor therapy. (**A**) Kaplan–Meier survival curves of GBM patients in the low expression and high expression subgroups of SLC1A5 following anti-PD-1 treatment in the PRJNA482620 cohort. (**B**) Kaplan–Meier survival curves of melanoma patients in the low expression and high expression subgroups of SLC1A5 following anti-PD-1 treatment in the GSE78220 cohort. (**C**) Kaplan–Meier survival curves of melanoma patients in the low expression and high expression subgroups of SLC1A5 following DC treatment in the GSE106128 cohort. (**D**) Kaplan–Meier survival curves of melanoma patients in the low expression and high expression subgroups of SLC1A5 following anti-CTLA-4 treatment in the Nathanson_2017 cohort. (**E**) Kaplan–Meier survival curves of renal cell carcinoma patients in the low expression and high expression subgroups of SLC1A5 following anti-PD-1 treatment in the Braun_2020 cohort. (**F**) Kaplan–Meier survival curves of renal cell carcinoma patients in the low expression and high expression subgroups of SLC1A5 following EVEROLIM treatment in the Braun_2020 cohort.
